# *Achillea* Species as Sources of Active Phytochemicals for Dermatological and Cosmetic Applications

**DOI:** 10.1155/2021/6643827

**Published:** 2021-03-25

**Authors:** Marcelina Strzępek-Gomółka, Katarzyna Gaweł-Bęben, Wirginia Kukula-Koch

**Affiliations:** ^1^Department of Cosmetology, Faculty of Medicine, The University of Information Technology and Management in Rzeszów, Sucharskiego 2, 35-225 Rzeszów, Poland; ^2^Department of Pharmacognosy, Medical University of Lublin, Chodźki 1, 20-093, Poland

## Abstract

*Achillea* spp. is well known for its broad range of applications and long history of use in traditional medicine around the world. Health benefits of *Achillea* extracts result from the multitude of secondary metabolites identified in the plants from this genus that include flavonoids, phenolic acids, terpenes, guaianolides, phytosterols, fatty acids, and organic acids. The properties of several *Achillea* extracts meet also the expectations of a vividly developing cosmetic market. An increasing number of studies on the dermatological properties of *Achillea* spp. are observed in the recent years, with *Achillea millefolium* L. being the most studied and used representative of the genus. There is strong scientific evidence showing that also other yarrow species might be rich sources of effective cosmetic ingredients, with skin calming and rejuvenating properties, wound healing activity, and anti-inflammatory potential. Several *Achillea* extracts and isolated compounds were also shown to display significant tyrosinase inhibitory, antioxidant, and antimicrobial properties and thus are interesting candidates for active ingredients of medications and cosmetic products protecting the skin from the harmful impact of environmental stressors. The aim of this review is to collect the current information on the composition and cosmeceutical significance of different *Achillea* species.

## 1. Introduction

The cosmetics market is one of the most dynamically developing markets in the world. The global increase in sales of skin care products in the past five years has been reaching 5.5% every year [1]. A growing interest in this branch of the economy is directly related to a raising consumers' awareness regarding the negative impact of various environmental factors on the skin condition, the desire to care for the body and delay the effects of skin aging that are fuelled by the social media and by the improvement of the material status of the societies. The main role of the active ingredients used in modern cosmetics is to protect the skin from air pollutants, ultraviolet radiation, and changing climate factors (temperature and wind) or reduce the irritation caused by the external factors and support regeneration mechanisms [2, 3]. Negative impact of the environmental stressors on the skin function has been strongly connected with an increased production of reactive oxygen species (ROS) and the generation of oxidative stress [4]. In view of the abovementioned data, there is a need to introduce to the market new cosmetic ingredients that will protect the skin constantly exposed to harmful environmental stressors from oxidative stress, reduce skin irritation and hyperpigmentation, and improve its regeneration and rejuvenation. Recent years have brought a return of plant-derived ingredients from organic plantations to medicines and cosmetics. The term “naturally derived” is well associated with consumers and is a guarantee of good sales of the product [5].

The constantly developing research on cosmetics and rising interest in high-quality skin care products encourage the manufacturers to use enriched extracts, fractions, or even single/purified components instead of crude extracts to achieve a better cosmetic effect. Resveratrol, caffeine, epigallocatechin gallate, and gallic acid are only few examples of purified natural products that are registered in the cosmetic ingredient database (CosIng). This trend, also visible in phytotherapy strategies, highlights the individual metabolites produced by plants as agents with the actual expected activity of the whole product.

The genus *Achillea* has a long history of use in traditional medicine as a natural remedy for the treatment of wounds, bleedings, headache, inflammation, pains, spasmodic diseases, flatulence, and dyspepsia. The health benefits of using *Achillea* extracts have been confirmed by numerous scientific studies and summarized in several recently published reviews [6–9]. *Achillea* extracts have been also used for long time as active ingredients of skin healing and skin conditioning products, but the application of *Achillea* in dermatology and skin care has not been reviewed do date. *Achillea millefolium* L. (common yarrow) extracts are the best known ingredients of cosmetic products among yarrow species, but current research data shows that also other *Achillea* species possess biological properties valuable for cosmetic and dermatology applications. Due to the broad distribution of these plants worldwide, extracts from other *Achillea* species may be easily accessible and effectively used as active ingredients of cosmetics and healing ointments. In order to summarize the current state of the knowledge regarding the dermatological and cosmetic properties of *Achillea* species for this review, a thorough research was performed in electronic databases, including PubMed, Scopus, and Web of Science. The search was performed using the term “*Achillea*” in combinations with the following terms: “antioxidant, skin, keratinocyte, melanogenesis, tyrosinase, elastase, collagenase, wound healing”.

In relation to the abovementioned trend in the introduction of enriched extracts or single molecules of plant origin to cosmetics, the aim of this review is to summarize the cosmetic significance of *Achillea* extracts and to describe the identity and properties of the active constituents that are responsible for the skin conditioning activity. Pure compounds isolated from *Achillea* are not used in cosmetic formulations currently, but the identification of their structures and properties might help to improve the production of *Achillea* extracts for dermatological and cosmetic applications.

## 2. Traditional Application of *Achillea* spp. in Skin Disorders

Species belonging to the genus *Achillea* are distributed worldwide, growing wild in Europe and temperate regions of Asia and North America. Due to a wide distribution of *Achillea* plants, they have been used since Middle Ages as natural remedies in several countries [10]. *Achillea millefolium* L. is certainly the most known and studied species of the genus, with the longest history of traditional applications. Two *Achillea* species—*A. millefolium* and *A. ageratum*—are described in an 18th-century Polish medical help book entitled *Compendium medicum auctum* as natural remedies, also for skin disorders [11]. Dried aerial parts of *A. millefolium* have been used as raw materials for the preparation of aqueous and alcoholic extracts applied externally in the form of compresses or baths for the treatment of skin and mucous membrane inflammations. Fresh or dried herb or freshly squeezed juice from *A. millefolium* leaves has been used in traditional European medicine to stop wound bleeding and promote healing of minor wounds, ulcerations, and sores. *A. millefolium* oil macerate, prepared by a three-hour-long maceration of fresh or dried herb in the vegetable oil, is recommended for the treatment of skin inflammation or as protection from the sunburns [6, 12].

*A. millefolium* has been used in folk medicine in Pakistan and Iran for wound healing. In Italy and Turkey not only *A. millefolium* but several other *Achillea* species extracts were traditionally administered as wound healing agents [13, 14]. Unani, medicine of India, concerned the activity of *A. millefolium* and described its significant role in traditional medicine and a successful administration in the treatment of inflammatory conditions and pain (wounds, cuts, and abrasions) that could be relieved by the application of lotions or ointments containing common yarrow extracts [7, 15]. Traditional Chinese medicine uses *A. millefolium* as an antihaemorrhagic, wound healing agent and as effective cosmetic soothing for sores, skin disorders (wounds), snakebites, and varicose veins [16]. In addition to *A. millefolium*, also *A. ligustica* has a long history of administration in Italian, Sicilian, and Sardinian folk medicine for the treatment of skin disorders, as anti-inflammatory and antimicrobial natural remedy [17].

## 3. Chemical Composition

Various biological effects of *Achillea* spp. may be explained by the presence of a wide range of natural products present in its extracts. The highest number of compositional studies was performed on *A. millefolium* so far. However, the scientific literature also shows the detailed composition of other representatives of the genus, e.g., *A. ligustica*, *A. clavennae*, *A. coarctata*, *A. kotschyi*, *A. monocephala*, and *A. pachycephala* (Table 1 and Table S1 in supplementary file).

*Achillea* spp. appear to be exceptionally rich in different types of secondary metabolites in comparison with other plant species. The most common compounds include flavonoids (glucosilated and nonglucosilated), phenolic acids (mostly cinnamic and benzoic acid derivatives), terpenes (including guaianolides, diterpens, sesquiterpenes, and their oxygenated forms), phytosterols, organic acids, fatty acids, and alcohols.

From the dermatological point of view, phenolic acids are an important group of metabolites spread in the extracts of *Achillea* plants. Several scientific publications mention the multitude of caffeic and quinic acid derivatives in various *Achillea* extracts. Among them (1,3-dicaffeoylquinic acid (1,3-DCQA), 1-feruloquinic acid, 3,4,5-tricaffeoylquinic acid (3,4,5-TCQA), 3,5-dicaffeoylquinic acid (3,5-DCQA), 3-caffeoylquinic acid (3-CQA), 4,5-dicaffeoylquinic acid (4,5-DCQA), 4-caffeoylquinic acid (4-CQA), 4-feruloquinic acid, 4-OH-benzoic acid, and 5-caffeoylquinic acid (5-CQA)) were identified. Also, the extracts contain several simple phenolic acids like ferulic, gallic, *o*-coumaric acid, *p*-coumaric acid, *p*-hydrobenzoic, protocatechuic, quinic, salicylic, sinapic, acid, *trans*-cinnamic, and vanillic acids [18–25] and vanillin—a phenolic aldehyde [23, 24].

Among the groups of metabolites identified in *Achillea* species, flavonoids are the most widely represented natural products in the extracts. They are present in the form of aglycones and glucosides. Keampferol and its glucosides (e.g., kaempferol-3-*O*-glucoside) are reported as the major components of *Achillea* extracts. Moreover, the scientists have confirmed the presence of other components from the same group: catechin, 5,7,3′-triacetoxy-3,6,4′-trimethoxyflavone and its isomer, 7-*O*-methyl apigenin, apigenin, apigenin-7-*O*-glucoside, axillarin, centaureidin, chrysosplenol B, fisetin, galetin 3,6-dimethyl ether, hesperetin, hesperidin, hyperoside, isoquercetin, isovitexin, jaceidin, kaempferol, kaempferol-3-*O*-glucoside, luteolin, luteolin-7-*O*-glycoside, luteolin-7-*O*-glucuronide, naringenin, nicotiflorin, penduletin, quercetin, and quercetin-3-*O*-glucuronide. The following flavonoid glucosides were indicated as the constituents of *Achillea* extracts: isoharmnetin-3-*O*-glucoside and isoharmnetin-3-*O*-rutinoside, isoschaftoside, neoschaftoside, and schaftoside [17, 19, 21–26].

Plants from this genus synthesize an interesting group of metabolites, namely, guaianolides. These phytochemicals were identified in *A. clavennae* (1-deoxy-1*α*-peroxy-rupicolin A, 1-deoxy-1*α*-peroxy-rupicolin B, and rupicolins A and B) and *A. millefolium* (8-hydroxyachillin, austricin, desacetylmatricarin dihydroparthenolide, dihydroreynosin, paulitin and its isomer, millefin, psilostachyin C, rupicolins A and B, syntenin, and *α*-peroxyachifolid) [7, 26].

The extracts from yarrow species are also rich sources of phytosterols. Stigmasterol and *β*-sitosterol were found in the extracts from the aerial parts of *A. ageratum*, *β*-sitosterol (in *A. ligustica*), and campesterol and cholesterol (in *A. millefolium*) in addition to the two previously mentioned compounds [7, 17, 24].

Several subgroups of terpenes were identified in *Achillea* species like hydrocarbon monoterpenes (camphene *α*, *cis*-chrysanthenol, limonene, myrcene, *p*-cymene, sabinene, *α*- and *β*-pinene, *β*-phellandrene, *γ*-terpinene, and *Δ*3-carene) [7, 17], oxygenated monoterpenes (1,8-cineole, (*E*)-chrysanthenyl acetate, borneol, bornyl acetate, camphor, carvacrol, carvone, cis-sabinene hydrate, dihydrocarveol, fenchyl acetate, hotrienol, linalool, myrcene, *p*-cymen-8-ol piperitone, santolina alcohol, terpinen-4-ol, *trans*-chrysanthenyl acetate, *trans*-carveol, *α*-terpineol, and *α*- and *β*-thujone) [4, 16], diterpenes ((*E*)-phytol, (*Z*)-phytol, and neophytadiene), sesquiterpene hydrocarbons ((*E*)-caryophyllene, (*Z*)-caryophyllene, aromadendrene isoledene, *α*-calacorene, *α*-selinene, and *γ*-gurjunene) [19], and oxygenated sesquiterpenes (*α*-cyperone and achillin) [17].

Hydrocarbons, like decane, dodecane, heptacosane, hexacosane, nonacosane, octacosane, pentacosane, tetracosane, triacontane, tricosane, and undecane, were also mentioned in the scientific literature [17].

Several compounds identified in various *Achillea* species were shown to possess significant dermatological or cosmetic properties. These compounds are summarized in Table 1.

## 4. Dermatological and Cosmetic Properties

According to the cosmetic ingredient database (CosIng) [29], only two *Achillea* species are currently used as sources of active ingredients for cosmetic formulations: *A. millefolium* and *A. asiatica*. Due to the presence of a broad range of metabolites, *A. millefolium* extracts display multifunctional properties as active ingredients of cosmetics, including cleansing, moisturizing, shooting, conditioning, masking, and refreshing functions (Table 2). The other *Achillea* ingredient found in cosmetic formulation available on the market is *Achillea asiatica* flower/leaf/stem extract used as hair and skin conditioning agent and humectant [29].

Despite limited applications of *Achillea* extracts on the cosmetic market, a wide range of recent research findings indicate that the extracts and compounds isolated from several *Achillea* species possess beneficial properties for the skin. Available scientific evidence suggests that *Achillea* species are easily accessible source of effective and safe compounds with potential application in cosmetics and topically applied ointments.

### 4.1. Skin Irritating Potential of *Achillea* Extracts

Introduction of a novel ingredient into the cosmetic formulation requires an assessment of the safety of its use. For that reason, each novel natural or synthetic ingredient undergoes determination of its irritating potential using various *in vitro* and *in vivo* methods. The most common and reliable assay used for the determination of irritating properties of a novel ingredient is the patch test. The patch test allows diagnosing contact allergy which results from the type IV hypersensitivity and eventually determines an allergic contact dermatitis. In this assay, tested components or prototypic cosmetic formulations are applied on a clean, dried, and unaffected area of the patient's body (the most often on the skin of the back or forearm) and kept for the following 48 hours. After the 2-day exposure, the test chambers are removed and the results are read usually after 15-60 minutes following chamber removal and also on the day 3 and day 4 [30, 31].

So far, the irritating potential was investigated only for the *A. millefolium* extracts among all yarrow species. None of the published *in vivo* and *in vitro* studies confirmed sensitizing or irritating potential of cosmetic formulations containing *A. millefolium*. In a human repeated insult patch test (HRIPT), a face moisturizer with self-tanner product containing *A. millefolium* extract (0.00045%; 0.2 ml) was not found irritating or sensitizing. There were transient, barely perceptible-to-mild nonspecific and specific responses, occasionally accompanied by mild/moderate oedema or mild dryness in 9 out of 107 tested participants. Five participants had mild hyperpigmentation without erythema during the induction phase [32]. In another HRIPT study (*n* = 108), a body splash product containing an extract of *A. millefolium* (0.001133%) was applied and found not irritating or sensitizing, as in the previous study. The HRIPT test (*n* = 53) of a body lotion containing *A. millefolium* extract (0.04%) confirmed the lack of irritation potential of *A. millefolium* as cosmetic ingredient. In a patch test performed in the participants with atopic dermatitis (*n* = 9), there were no positive reactions to *A. millefolium* extract (1% in petrolatum) [33].

Irritating potential of *A. millefolium* extract (0.00045%) was also assessed using a 3D model of human cornea EpiOcular. This analysis confirmed the safety of *A. millefolium* extract as an ingredient of around-eye cosmetics. Based on the available data, *A. millefolium* extract, *A. millefolium* flower extract, and *A. millefolium* flower/leave/steam extracts are considered as safe ingredients of cosmetic formulations [32].

**Figure 1:**
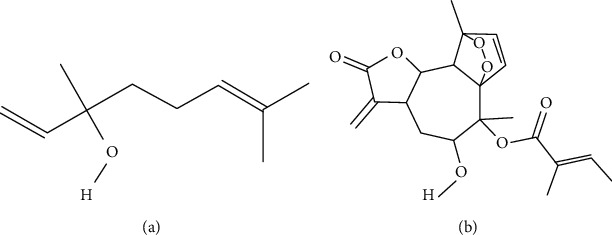
Examples of potential dermal sensitizers identified in volatile fraction of *A. millefolium* plant: linalool (a) and *α*-peroxyachifolid (b).

Despite the fact that the total *A. millefolium* extracts themselves did not provide any evidence on the skin irritating properties, the authors of the safety report pointed out the single components of the volatile fraction of the *A. millefolium* plant may raise safety concerns. Among them, linalool and *α*-peroxyachifolid (Figure 1) are confirmed dermal sensitizers, thujone was reported to cause neurological toxicity upon an oral intake, and quercetin was proved to have some genotoxic effects in *in vitro* assays [32]. The knowledge about the content of potentially irritating and toxic substances in the plant material helps in planning the proper extraction procedure, resulting in a safe to use extract rich in compounds improving skin condition.

### 4.2. Skin Calming and Anti-Inflammatory Activities

Application of *Achillea* extracts is recommended for sensitive skin type, defined by sensory symptoms like burning, prickling, and tingling due to the various external factors including cold, heat, water, wind, pollution, UV radiation, or the use of inappropriate cosmetics. Occasionally, these subjective symptoms are accompanied by erythema [34]. *Achillea spp*. are used traditionally in order to tread skin irritation and exhibit skin calming activity that was proved experimentally. An *in vivo*, double-blind, randomized study showed significant skin calming and anti-inflammatory potential of *A. millefolium* sunflower and olive oil macerates, with 8% (*v*/*v*) sodium lauryl sulfate (SLS) as artificial irritant. The skin parameters assessed in this study (skin capacitance, pH, and erythema index) were restored to the basal values after three- and seven-day treatment [12].

Inflammatory diseases are the most common problems in dermatology. Inflammation process results from the disruption of the skin barrier and following activation of innate and adaptive immune responses [35]. During inflammation, several soluble agents are produced and secreted by the skin cells and specialized immune cells present in the skin, activating signaling pathways of different types. Inflammatory mediators are usually divided into two main categories: anti-inflammatory and proinflammatory agents. Anti-inflammatory properties of several natural and synthetic agents usually depend on their inhibition of proinflammatory cytokines, for example, tumour necrosis factor-*α* (TNF-*α*) and interleukin-1 (IL-1), IL-6, and IL-10, and an induction of anti-inflammatory compounds, such as transforming growth factor-*β* (TGF-*β*) and IL-10 [36].

Anti-inflammatory properties of *A. millefolium* are well known from both traditional applications and also experimental evidence. A polysaccharide fraction called Am-25-d, isolated from the aqueous extract of *A. millefolium*, was shown to increase lipopolysaccharide- (LPS-) induced secretion of IL-1*β*, IL-8, IL-10, IL-12p40, IL-23, and TNF-*α* cytokines by human monocyte cell line THP-1 activated with interferon-*γ* (INF-*γ*) and LPS. Moreover, THP-1 cells cultured in the presence of Am-25-d decreased nuclear concentrations of proinflammatory nuclear factor kappa NF-*κ*B and phosphorylation of ERK1/2 and Akt kinases when compared to the cells cultured without polysaccharide fraction. Based on these results, it was concluded that polysaccharide fraction Am-25-d isolated from *A. millefolium* has immune-enhancing properties that may be mediated via the Akt kinase pathway [37].

In order to obtain *A. millefolium* extracts with increased anti-inflammatory properties, supercritical antisolvent fractionation (SAF) was applied to ethanolic extract. THP-1 cells were activated with LPS in the presence of 5 and 10 *μ*g/ml of crude extract and SAF fractions. Obtained results allowed to identify fractions with the most significant anti-inflammatory potential (decreased TNF-*α*, IL-1*β*, and IL-6 secretion in comparison with the cells treated with LPS only), indicating that SAF may be used to partially separate *A. millefolium* compounds with high anti-inflammatory potential [38].

Anti-inflammatory activity of *A. millefolium* may benefit atopic dermatitis patients as demonstrated by Ngo and coworkers. In this study, 50% ethanol extracts from *A. millefolium* were analyzed for their anti-inflammatory activity using three models corresponding to atopic dermatitis condition: RAW 264.7 macrophages, HaCaT immortalized human keratinocytes, and Biostir-AD-treated NC/Nga mice in vivo. *A. millefolium* extract significantly downregulated expression of proinflammatory cytokines such as IL-6, isoform nitric oxide synthase (iNOS), and cyclooxygenase-2 (COX-2) in LPS-treated RAW 264.7 cells. In HaCaT cells stimulated with INF-*γ* and TNF-*α*, *A. millefolium* extract treatment markedly decreased expression of proinflammatory chemokines and TNF-*α* through the activation of the MAPK and STAT-2 signaling pathways. Finally, dietary administration of *A. millefolium* extract to Biostir-AD-treated NC/Nga mice reduced atopic dermatitis symptoms including elevated serum immunoglobin E (IgE) levels, epidermal thickening, high dermatitis severity score, transepidermal water loss, and reduced skin hydration. Presented results suggest that *A. millefolium* extracts may be used for atopic dermatitis treatment [39]. Despite a great amount of scientific evidence confirming the anti-inflammatory properties of *A. millefolium*, active compounds directly responsible for the multiple anti-inflammatory reactions were not identified to date.

**Figure 2:**
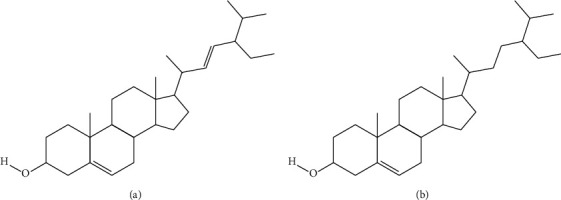
*A. ageratum* compounds with skin calming activity: stigmasterol (a) and *β*-sitosterol (b).

Among other *Achillea* species, *A. ageratum* is the most interesting source of anti-inflammatory compounds. Chloroform extracts from the aerial parts of this plant reduced myeloperoxidase (MPO) activity and oedema in 12-0-tetradecanoylphorbol acetate- (TPA-) induced mouse ear oedema model, using simple (acute model) and multiple applications (chronic model) of the phlogistic agent. Stigmasterol and *β*-sitosterol (Figure 2), isolated from the chloroform extract, were shown to participate in the observed calming effect [27]. Both compounds were previously proved to be effective anti-inflammatory agents in topical application [40].

### 4.3. Wound Healing Potential

A wound can be defined as a damage or disruption to the normal anatomical structure of the skin and its functions. Wounding damages the tissue and destroys a local environment and physiological processes. The wound healing is a long-term process which includes bleeding, coagulation, regeneration, migration, and proliferation of connective tissue and parenchyma cells, remodelling of new parenchyma, connective tissue, and collagen [41–43]. Fibroblasts play a beneficial role during healing process. Following tissue injury, fibroblasts infiltrate and degrade the fibrin clot by producing various matrix metalloproteinases (MMPs), replacing it with extracellular matrix (ECM) components, such as collagen, glycoproteins, proteoglycans, laminin, thrombospondin, glycosaminoglycans (GAGs), hyaluronic acid (HA), and heparan sulfate. The complex matrix supports and regulates the migration and activity of fibroblasts, as well as provides support and signals for angiogenesis, granulation tissue generation, and epithelialization [44].

**Figure 3:**
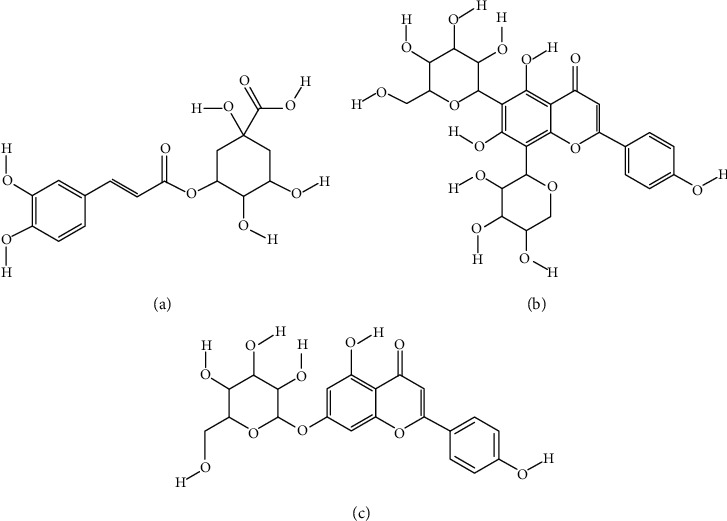
Compounds from *A. asiatica* responsible for the wound healing and reduced scar formation activities: chlorogenic acid (a), schaftoside (b), and apigenin-7-*O*-glucoside (c).

Several scientific publications indicate that *Achillea* spp. accelerate would healing and reduce scar formation. Hydroalcoholic extracts (ethanol: water 8 : 2, *v*/*v*) from aerial parts of *A. millefolium* (leaves, stems, and stalks) at the concentrations below 20 mg/ml were shown to increase proliferation of human skin fibroblast cells HFS-PI-16 in vitro and to stimulate wound closure in an in vitro scratch assay [45]. In the Sprague-Dawley rat model, the extract from the aerial parts of *A. asiatica* was shown to enhance the wound closure rate starting from day 8 and to reduce the wound area by approximately 50% comparing to an untreated control wound. The molecular mechanism of *A. asiatica* action in wound healing was explained by *in vitro* experiments showing both the extract and its constituents (chlorogenic acid and schaftoside) as stimulators of Akt phosphorylation and inducers of nuclear translocation of *β*-catenin in keratinocytes. These mechanisms triggered the migration of keratinocytes to the wound site and initiated the differentiation process. The fractions obtained from the ethanol extract of *A. asiatica* collected in Mongolia were studied for their impact on the viability of human dermal fibroblast Hs68 and human keratinocytes HaCaT and the role in the wound healing process in rats. *A. asiatica* extract reduced the wound area in comparison with positive control (*Centella asiatica* extract). *A. asiatica* induced keratinocyte migration to the wound via the activation of the *β*-catenin, ERK, and Akt signaling pathways and initiated the process of keratinocyte differentiation. Three compounds identified in the extract (apigenin-7-*O*-glucoside, schaftoside, and chlorogenic acid) (Figure 3) were found to be responsible for the observed wound healing activity. Also, the total *A. asiatica* extract increased the expression of collagen I gene, whereas schaftoside enhanced the expression of collagen III gene [21].

Another *Achillea* species with a confirmed wound healing activity is *A. biebersteinii*. The extracts from this plant possess significant wound healing properties, confirmed by *in vitro* and *in vivo* studies. The *in vivo* studies performed in the Sprague-Dawley rats and Swiss albino mouse animal models included the treatment of wounds with *A. biebersteinii* formulation in comparison with commercially available wound healing drug Madecassol. In the study, the propylene glycol : liquid paraffin (6 : 1) ointment, containing 1% n-hexane extract from *A. biebersteinii*, increased wound contraction by 84.2%, which was very close to the standard drug (100% contraction). Also, the same ointment demonstrated a significant increase (40.1%) in wound tensile strength in the incision wound model as compared to control animals [46]. Hydroalcoholic extracts from the flowers of *A. biebersteinii* (5 *μ*g/ml and 10 *μ*g/ml, 12- and 24-hour treatment) may inhibit scar formation as they downregulated the expression of the transforming growth factor beta 1 (TGF-*β*1) and upregulated the expression of the basic fibroblast growth factor (bFGF) on gene and protein levels in murine embryonic fibroblasts *in vitro* [47]. TGF-*β*1 may mediate fibrosis in wound, while bFGF may promote scarless healing and reduce scarring in this process [48]. Methanol extract from the leaves of *A. biebersteinii* was not cytotoxic to human foreskin fibroblasts HFF3 at the concentration range of 1-512 *μ*g/ml, during 24 h, 48 h, and 72 h of treatment, indicating potential safety of its application [49].

Wound healing properties were also demonstrated by at least four other *Achillea* species. Aqueous extract from the flowers of *A. kellalensis* showed an increased wound healing activity in rats. The wound sizes were reduced upon topical administration of the extract when compared to the control groups [50]. Methanolic extracts from *A. coarctata* (AC), *A. kotschyi* (AK), and *A. lycaonica* (AL) were studied for wound healing properties by Agar and coworkers using an *in vitro* murine fibroblast cell line: NIH-3T3. In the study, the AK extract increased the total fibroblast proliferation at the concentrations of 2.5 *μ*g/ml, 5 *μ*g/ml, 10 *μ*g/ml, and 20 *μ*g/ml. Upon its administration, a decreased number of quiescent fusiform cells were observed together with an increased percentage of active polygonal fibroblasts. According to the results, AK could stimulate the migration of fibroblasts to the wounds. As an indicator of a noncytotoxic effect of AK, the percentages of round fibroblasts and vacuole containing cells were found to be strongly decreased. What is more, AK extract markedly stimulated the production of collagen in the NIH-3T3 cells at low concentrations. Similar impact of AC and AL extracts on collagen synthesis was observed at a concentration of 10 *μ*g/ml, 5 *μ*g/ml, and 10 *μ*g/ml of AC- and AL-stimulated fibroblast [23]. Phytochemicals responsible for the wound healing activity of *A. biebersteinii*, *A. kellalensis*, *A. coarctata*, *A. kotschyi*, and *A. lycaonica* have not yet been identified.

### 4.4. Skin Rejuvenation

The process of human skin aging is characterized by an increased skin laxity, appearance of visible lines and wrinkles, and an overall deterioration of the skin texture. Skin rejuvenation effect refers to the achievement of a better organized dermal matrix and epidermal structure, resulting in a smoother and more tense and tone skin [51]. Skin rejuvenating activity was shown mostly for *A. millefolium* extracts. Aqueous extracts from this species induced both mRNA and protein expressions of melanocortin receptor-2 (MC-2R) and l-opioid receptor-1 (MOR-1) in human keratinocyte *in vitro* cultures. The expression of these two receptors for adrenocorticotrophic hormone (ACTH) and *β*-endorphin decreases with aging in normal human epidermis and is considered to be responsible for the structural and functional alterations of human epidermis. The treatment of normal human epidermal biopsies *ex vivo* with *A. millefolium* extracts induced the expression of epidermal differentiation markers (cytokeratin 10, transglutaminase-1, and filaggrin) and increased epidermal thickness by 10%. Moreover, *in vivo* study showed that a two-month treatment with 2% *A. millefolium* extract significantly improved the appearance of wrinkles and pores compared with placebo. The observed skin rejuvenating effect was also more pronounced than that of glycolic acid, a reference resurfacing and rejuvenating molecule [52].

### 4.5. Antimicrobial Activity

One of the most important activities of ingredients used in cosmetics and dermatological ointments is antimicrobial activity. Microorganisms, such as bacteria and fungi, create a natural microflora on the human skin, but species may cause serious infections and participate in skin disorders such as acne vulgaris or skin mycosis [53]. The most important microorganisms causing skin infections include *Staphylococcus aureus*, partially responsible for atopic dermatitis (AD) symptoms. The degree of colonization significantly correlates with the intensity of the disease [54]. *S. aureus* induce superficial infection of the epidermis (impetigo), deep ulcerative skin infections, infection of hair follicles, abscesses, wounds, and ulcers [55]. The occurrence of *Pseudomonas aeruginosa* infections is typical in burns, surgical wounds, and vascular diseases. Among other methods of infection mechanical ventilation, immunodeficiency and chronic pulmonary disorders should be listed. Based on long-term observations, it was concluded that infection of *P. aeruginosa* is favoured by the broken epidermis [56]. Among fungi, *Candida* species are those that most frequently cause chronic mucocutaneous candidiasis characterized by chronic, persistent, and mucocutaneous lesions [57]. Also, it is worth to note that *P. aeruginosa*, *S. aureus*, and *C. albicans* are considered the main potential pathogens in cosmetic products. Plant extracts that are active antimicrobial or antifungal agents are precious, as they can reduce the skin infections and additionally inhibit the growth of microorganisms inside cosmetic products [58].

Among *Achillea* species, *A. millefolium* extracts are the most potent antimicrobial ingredients that were proved to inhibit the pathogens causing skin infections, including *S. aureus* and also usually resistant to plant extracts—*P. aeruginosa* [18]. *A. biebersteinii* extracts were also found to inhibit the growth of pathogens causing dermal infections such as *S. aureus* with minimal inhibitory concentration (MIC) of 0.6 ± 0.004 mg/ml, 0.1 ± 0.005 mg/ml, and 0.2 ± 0.006 mg/ml for water, methanol, and ethyl acetate extracts, respectively. The extracts were also suppressing the growth of *P. aeruginosa* (MIC = 0.3 ± 0.005 mg/ml for water extract, 0.1 ± 0.003 mg/ml for methanol extract, and 0.1 ± 0.003 mg/ml for ethyl acetate extract) [18]. This activity indicates its potential application as an antibacterial active ingredient and also natural preservative of cosmetic formulations.

**Figure 4:**
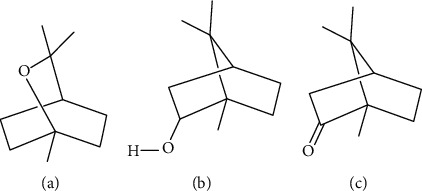
Compounds identified in *A. ligustica* and *A. millefolium* which exhibited antimicrobial properties: 1,8-cineole (a), borneol (b), and camphor (c).

Also, the essential oils obtained from the flowers and leaves of *A. ageratum* were proved to possess antibacterial activity against *S. aureus* (MIC = 27 − 29.1 mg/ml) and *C. albicans* strains (MIC = 15.7 − 34.8 mg/ml) [59]. In the study by Zengin and coinvestigators, also *A. teretifolia* inhibited *S. aureus* (MIC = 0.15 ± 0.001 and 0.10 ± 0.003 mg/ml for methanol and ethyl acetate extracts, respectively) and *P. aeruginosa* (MIC = 0.05 ± 0.001 mg/ml and 0.10 ± 0.05 mg/ml for methanol and ethyl acetate extracts, respectively) [18]. Compounds responsible for the observed antibacterial effect against pathogens causing dermal infections were not identified to date. However, compounds such as 1,8-cineole, borneol, and camphor, identified for example in *A. ligustica* and *A. millefolium*, are known for their antibacterial activity (Figure 4) [7, 17, 60].

### 4.6. Antioxidant Activity

Oxidative stress plays an important role in the undesirable biochemical pathways of the human body. It is defined as an imbalanced status between the production of reactive oxygen species (ROS) and the physiological system of enzymes and nonenzymatic compounds responsible for the neutralization of ROS. Free radicals and other reactive oxygen molecules are responsible for the disruption of DNA, lipids, proteins, and carbohydrates, leading to the premature aging and the development and progression of cancer, including skin cancer. ROS play an important role in carcinogenesis through two possible mechanisms: the induction of gene mutations and the effects on signal transduction and transcription factors. Delivery of antioxidants—the compounds which can inhibit the production of reactive oxygen species and limit their propagation—is currently one of the major aspects of cancer chemoprevention [61–63].

*Achillea* extracts are rich sources of flavonoids, which their glucosides and phenolic acids are known as the strongest antiradical metabolites. Several studies have been conducted so far on *Achillea* extracts and their metabolites that confirm strong antioxidant properties of the representatives of this genus.

**Figure 5:**
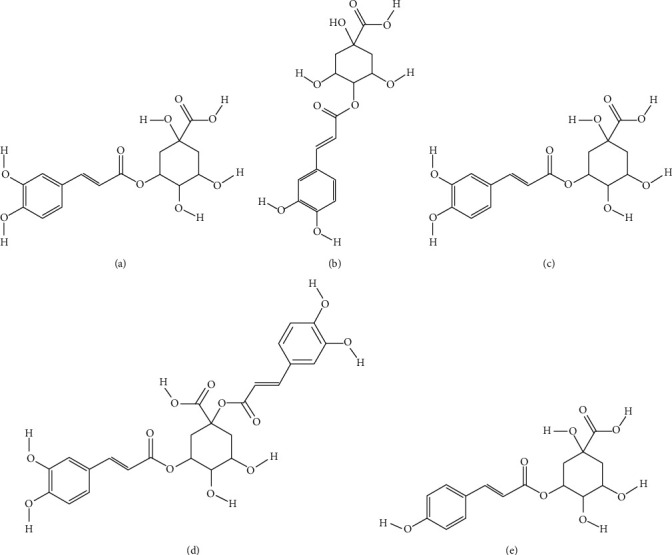
*Achillea* spp. compounds with significant antioxidant activity: 3-caffeoylquinic acid (a), 4-caffeoylquinic acid (b), 5-caffeoylquinic acid (c), 1,3-dicaffeoylquinic (d), and p-coumaroylquinic acid (e).

One of the most prominent antioxidant phytochemicals found in extracts from several *Achillea* species are caffeoylquinic acid derivatives. *Achillea* extracts rich in 3-caffeoylquinic acid, 5-caffeoylquinic acid, 4-caffeoylquinic acid, coumaroylquinic acid isomers, and 1,3-dicaffeoylquinic acid (Figure 5) were shown to possess significant antioxidant potential [64, 65]. Methyl 3,5-dicaffeoylquinic acid isolated from *A. alpina* was shown as effective antioxidant molecule using three independent methods—DPPH (2,2-diphenyl-1-picrylhydrazyl) scavenging assay, ABTS (2,2′-azino-bis(3-ethylbenzothiazoline-6-sulfonic acid) neutralization assay, and superoxide dismutase (SOD) activity assay. The obtained IC_50_ values for this compound were as follows: 60.66 ± 2.28 *μ*M in DPPH assay, 98.17 ± 4.78 *μ*M in ABTS assay, and 32.88 ± 3.36 *μ*M for SOD assay [22].

The antioxidant properties of water, methanol, and ethyl acetate extracts from 3 different species of *Achillea* species grown in Turkey (*A. biebersteinii*, *A. millefolium*, and *A. teretifolia*) were assessed by Zengin and collaborators. In this study, the ethyl acetate extracts were found the most rich in antioxidants. DPPH method proved that *A. biebersteinii* methanolic extract had the highest antioxidant capacity (126.9 mg of Trolox equivalents (TE)/g extract). *A. millefolium* water extract exhibited the highest antioxidant activity in TEAC (Trolox equivalent antioxidant capacity) assay (518.1 mg TE/g extract), and ethyl acetate extracts from *A. teretifolia* were characterized by the highest values of chelating activity against Fe^2+^ (20.64 mg EDTA equivalents/g extract); however, a significant chelating activity was exhibited by all extracts. FRAP (ferric reducing antioxidant power) assay selected *A. biebersteinii* methanolic extract as the strongest antioxidant, whereas CUPRAC (cupric reducing antioxidant capacity) method confirmed the s**t**rongest antioxidant capacity of *A. millefolium* methanolic extract (255.66 mg TE/g extract) [15]. Methanol leaf extract of *A. biebersteinii* effectively scavenged free radicals in DPPH, BCB (*β*-carotene bleaching), and TBARS (thiobarbituric acid reactive substances) tests, with IC_50_ of 0.27, 0.16, and 13.96 mg/ml, respectively. Moreover, pretreatment of HFF3 fibroblasts with 1 *μ*g/ml *A. biebersteinii* methanol extract protected the cells from H_2_O_2_-induced oxidative damage and prevented oxidative DNA damage, as shown by the comet assay [49]. Significant antioxidant activity of *A. biebersteinii* methanol extract was also confirmed by others, using DPPH scavenging, FRAC, and CUPRAC methods. The extract showed also significant Fe^2+^ chelating activity [18].

*A. monocephala* exhibited beneficial antioxidant properties when compared with *α*-tocopherol as positive control (IC_50_ = 16.85 ± 0.60 *μ*g/ml for DPPH method and IC_50_ = 5.41 ± 0.25 *μ*g/ml for ABTS assay) [24]. Another study describes a marked antioxidant potential of *A. phrygia* assessed using DPPH scavenging assay (IC_50_ = 109.34 mg TE/g for methanolic extract) and ABTS scavenging test (350.55 mg TE/g for water extract) [16]. Also, *A. kotschyi* showed an antioxidant potential in a DPPH radical scavenging assay (EC_50_ = 32.63 ± 0.65 *μ*g/ml) and in TAC (total antioxidant capacity) assay (2.080 ± 0.064 mM UAE) [23].

### 4.7. Skin Lightening Activity

**Figure 6:**
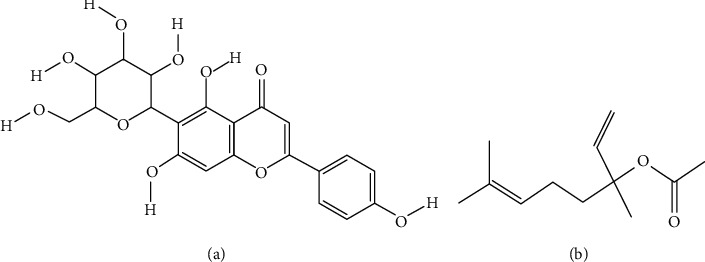
Isovitexin (a) and linalyl acetate (b)—skin lightening compounds found in *Achillea* species.

Pigmentation disorders, defined as localized or uneven distribution of melanin pigment, are currently one of the major targets of modern cosmetics or dermatological treatments. Novel, safe, and effective skin lightening agents are in constant need due to the serious concerns regarding the safety of hydroquinone, arbutin, and kojic acid, currently used in this type of cosmetics and dermatological therapies [66]. The main target of the skin lightening ingredients is tyrosinase (EC.1.14.18.1), a rate-limiting enzyme of the melanogenesis process. Tyrosinase catalyzes the conversion of L-tyrosine to L-dihydroxyphenylalanine (L-DOPA) (monophenolase activity) and subsequently to dopaquinone (diphenolase activity) [67]. Inhibitory studies using mushroom tyrosinase or murine melanoma cell line B16F10 were the most commonly used experimental models evaluating skin lightening potential of *Achillea* extracts and compounds. *A. millefolium* ethyl acetate, methanol, and water extracts have potential skin lightening activity as they inhibit mushroom tyrosinase *in vitro* with IC_50_ values of 31.57, 23.26, and 15.23 mg of kojic acid equivalents (KAE)/g, respectively [18]. Essential oil of *A. millefolium* suppressed melanin production in *α*-MSH-treated B16F10 melanoma cells through the regulation of the JNK and ERK signaling pathways. Linalyl acetate (Figure 6) was identified as the major active compound of *A. millefolium* essential oil responsible for this activity [28]. Isovitexin (Figure 6), compound isolated from *A. alpina*, downregulated the production of melanin in B16F10 melanoma cells [22]. Significant tyrosinase inhibitory activity was detected in water and methanol extracts from the aerial parts of *A. phrygia* (31.25 mg KAE/g and 23.06 mg KAE/g, respectively) [19]. Moderate tyrosinase inhibitory activity (23.42–28.28%) was also shown for ethanol extract from roots and aerial parts of *A. coarctata* and roots of *A. monocephala* [21]. In a study comparing tyrosinase inhibitory properties of *A. millefolium*, *A. biebersteinii*, and *A. teretifolia* water, methanol, and ethyl acetate extracts, the highest inhibitory effect of tyrosinase was observed for *A. biebersteinii* methanol extract (34.24 mg KAE/g) and ethyl acetate extract from *A. teretifolia* (34.18 mg KAE/g) [18]. Recently, significant tyrosinase inhibitory properties were also described for hydroglycolic extracts from *A. biebersteinii* and *A. millefolium*, inhibiting both monophenolase and diphenolase activity of this enzyme [68].

## 5. Future Perspectives

Currently, only two *Achillea* species—*A. millefolium* and *A. asiatica*—are used as active ingredients of cosmetic products. However, as proven in this review, the *Achillea* genus contains several species with a high potential application in medicinal and cosmetic products improving a broad range of skin conditions (Table 3).

A multitude of studies on various *Achillea* species and their effect on the skin that are presented above underline the importance of this genus in terms of future application in dermatology and cosmetics.

It is worth to note that the different species of yarrow are interesting sources of secondary metabolites of different kinds and there is still a lot to discover in terms of the application of single constituents from these plants. As described above, phenolic acids, flavonoids, terpenes, sterols, guaianolides, and esters of fatty acids are highly represented in their extracts. The activity of these precious molecules from the cosmetic point of view certainly needs further evaluation. Toxicity studies, human studies, and 3D skin model-based tests should follow the results from the herein described *in vitro* studies to show eventual toxicity and confirm the effects on living organisms.

## Figures and Tables

**Table 1:** Secondary metabolites of *Achillea* genus with known dermatological or cosmetic properties.

Compound	*Achillea ageratum*	*Achillea alpina*	*Achillea asiatica*	*Achillea aucheri*	*Achillea biebersteinii*	*Achillea coarctata*	*Achillea filipendulina*	*Achillea kotschyi*	*Achillea ligustica*	*Achillea lycaonica*	*Achillea millefolium*	*Achillea monocephala*	*Achillea moschata*	*Achillea nobilis*	*Achillea pachycephala*	*Achillea phrygia*	*Achillea santolina*	*Achillea teretifolia*
1,3-DCQA				[19]	[18]		[19]				[18], [19]			[19]	[19]		[19]	[18]
1,8-Cineole									[17]		[7]							
3-CQA					[18]						[18], [19]							[18]
4-CQA					[18]						[18], [19]							[18]
5-*O*-CQA		[22]											[22]					
*α*-Peroxyachifolid											[7]							
*β*-Sitosterol	[27]								[17]		[7]							
p-CQA											[7]							
Apigenin-7-*O*-glucoside			[21]										[25]					
Borneol									[17]		[7]							
Camphor									[17]		[7]							
Chlorogenic acid		[22]	[21]	[19]		[23], [24]	[19]	[10]	[17]	[23]	[19]	[24]		[19]	[19]	[20]	[19]	
Isovitexin		[22]																
Linalool											[7]							
Linalyl acetate											[28]							
Schaftoside		[22]	[21]															
Stigmasterol	[27]										7							

All numbers in the table resemble the scientific literature position from the References section that confirms the presence of a given metabolite in *Achillea* extracts. DCQA: dicaffeoylquinic acid; CQA: caffeoylquinic acid.

**Table 2:** Cosmetic ingredients from *Achillea millefolium* [29].

INCI name	Description	Function in cosmetic formulation
*Achillea millefolium* extract	Extract from the leaves and flowers	Antidandruff, cleansing, masking, refreshing, skin conditioning, shooting, tonic
*Achillea millefolium* flower extract	Extract from the flowers	Antioxidant, humectant
*Achillea millefolium* flower water	Aqueous solution from the steam distillated obtained from the flowers	Masking
*Achillea millefolium* flower/leaf/stem juice	Juice pressed from the flowers, leaves, and stems	Skin conditioning
*Achillea millefolium* oil, yarrow oil	Essential oil obtained from the flowering herb	Antidandruff, cleansing, masking, perfuming, refreshing, shooting, tonic

**Table 3:** Biological effects of *Achillea* species relevant for dermatological medicines and cosmetic products.

Activity	*Achillea* species	Ref.
Skin calming and anti-inflammatory properties	*Achillea millefolium*	[12], [37], [38], [39]

Wound healing properties	*Achillea asiatica*	[21]
	*Achillea biebersteinii*	[46], [47]
	*Achillea coarctata*	[23]
	*Achillea kellalensis*	[12]
	*Achillea kotschyi*	[23]
	*Achillea lycaonica*	[23]
	*Achillea millefolium*	[45]

Skin rejuvenating activity	*Achillea millefolium*	[53]

Antimicrobial properties against *P. aeruginosa*, *S. aureus*, and *Candida spp.*	*Achillea ageratum*	[59]
	*Achillea biebersteinii*	[18]
	*Achillea millefolium*	[18]
	*Achillea teretifolia*	[18]

Antioxidant properties	*Achillea alpina*	[22]
	*Achillea biebersteinii*	[18]
	*Achillea kotshyi*	[23]
	*Achillea millefolium*	[18]
	*Achillea monocephala*	[24]
	*Achillea phrygia*	[20]
	*Achillea teretifolia*	[18]

Skin lightening effect	*Achillea alpina*	[22]
	*Achillea biebersteinii*	[18], [68]
	*Achillea coarctata*	[23]
	*Achillea millefolium*	[18], [68]
	*Achillea monocephala*	[24]
	*Achillea phrygia*	[20]
	*Achillea teretifolia*	[18]

## Data Availability

No datasets were generated or analyzed during the current study.

## Supplementary Materials

Supplementary Materials See Table S1 in the Supplementary Material for comprehensive image analysis.
