# Supplementary material for: Achillea Species as Sources of Active Phytochemicals for Dermatological and Cosmetic Applications
Source: Oxid Med Cell Longev. 2021 Mar 25;2021:6643827. doi: 10.1155/2021/6643827 (PMC8018854; doi:10.1155/2021/6643827)
Supplement: Supplementary Materials — See Table S1 in the Supplementary Material for comprehensive image analysis. [file 6643827.f1.docx]

**Supplementary file**

**Oxidative Medicine and Cellular Longevity**

***Achillea* species as sources of active phytochemicals for dermatological and cosmetic applications – a review**

Marcelina Strzępek-Gomółka,^1^ Katarzyna Gaweł-Bęben,^1^ and Wirginia Kukula-Koch^2^

^1^ Department of Cosmetology, Faculty of Medicine, The University of Information Technology and Management in Rzeszów, Sucharskiego 2, 35-225 Rzeszów, Poland.
^2^ Chair and Department of Pharmacognosy, Medical University of Lublin, Chodźki 1, 20-093 Poland.

Correspondence should be addressed to Katarzyna Gaweł-Bęben; kagawel@wsiz.edu.pl and Wirginia Kukula-Koch; virginia.kukula@gmail.com

**Table S1**. The chemical structures of the identified constituents of *Achillea* extracts


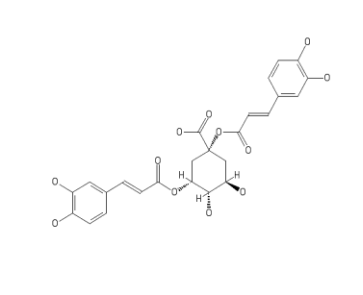


1,3-dicaffeoylquinic

acid


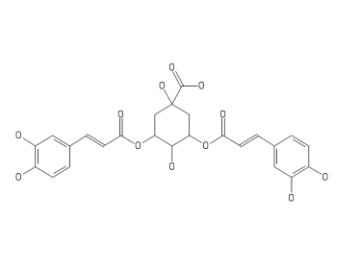


3,5-

dicaffeoylquinic

acid


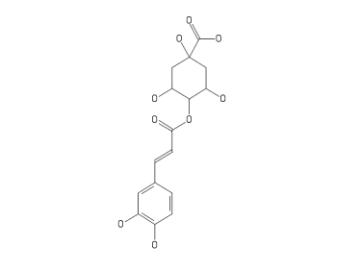


4-caffeoylquinic

acid


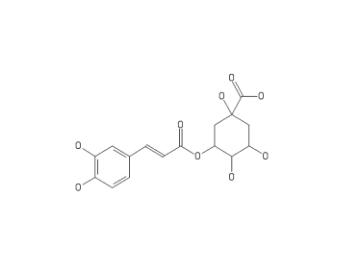


5-*O*-caffeoylquinic acid


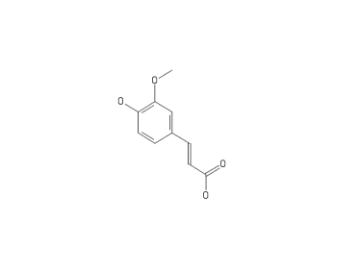


**Phenolic acids**

**
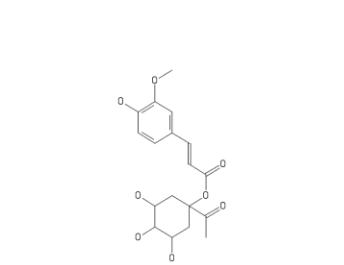
**

1-feruloquinic acid


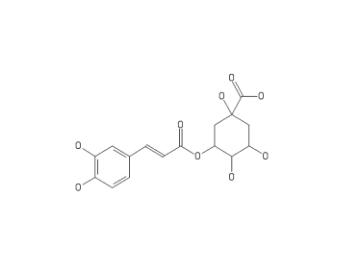


3-caffeoylquinic

acid


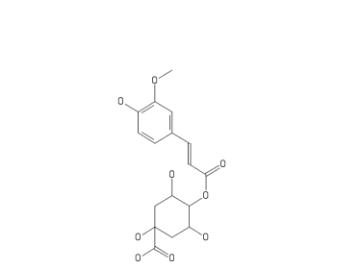


4-feruloquinic acid


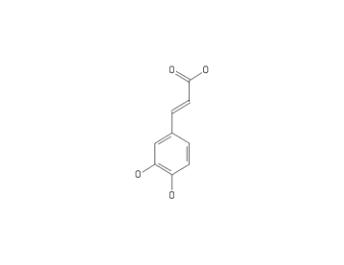


Caffeic acid


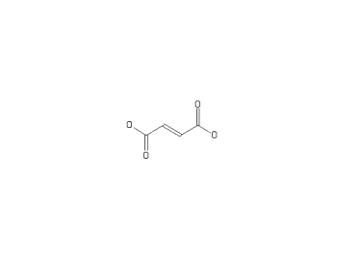


3,4,5-


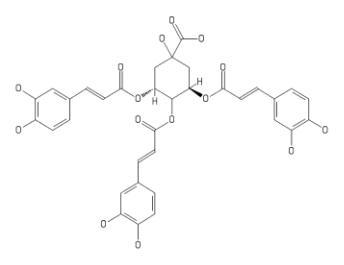


tricaffeoylquinic

acid


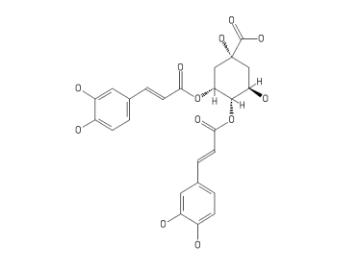


4,5-dicaffeoylquinic acid


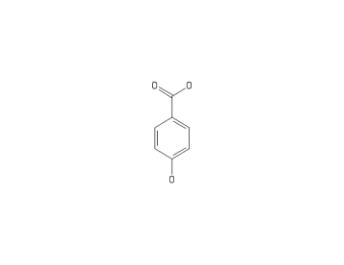


4-OH-Benzoic

acid


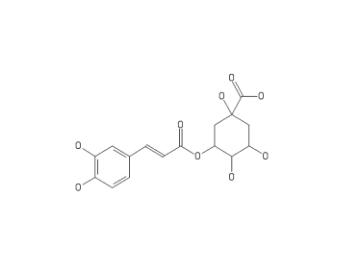


Chlorogenic acid


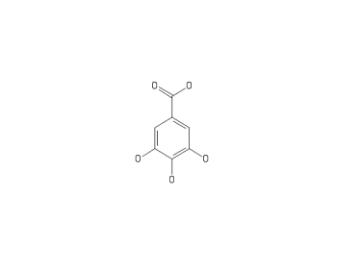


Ferulic acid Fumaric acid Gallic acid


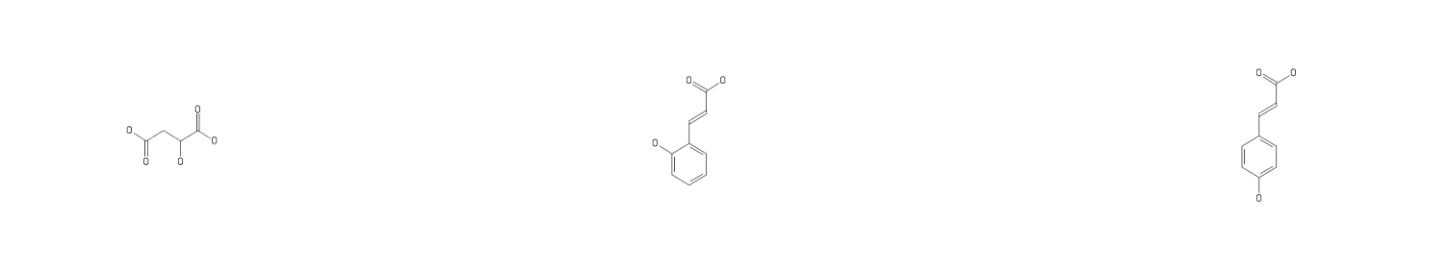


Malic acid *O*-coumaric acid *P*-coumaric acid


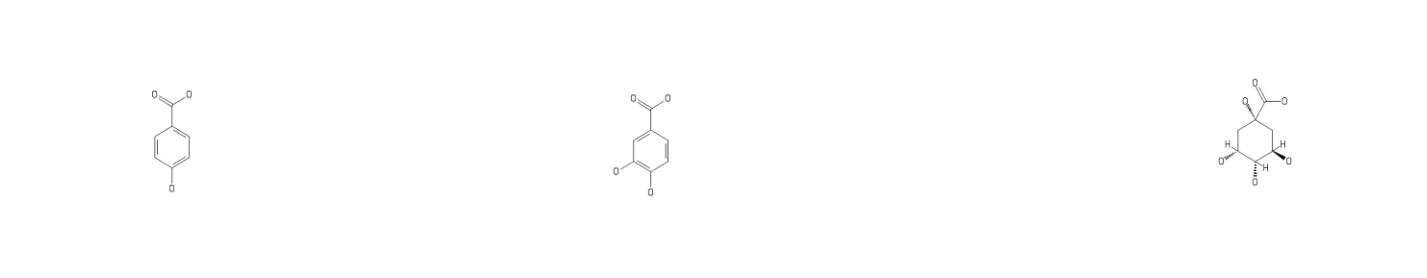


*P*-hydrobenzoic acid Protocatechuic acid Quinic acid


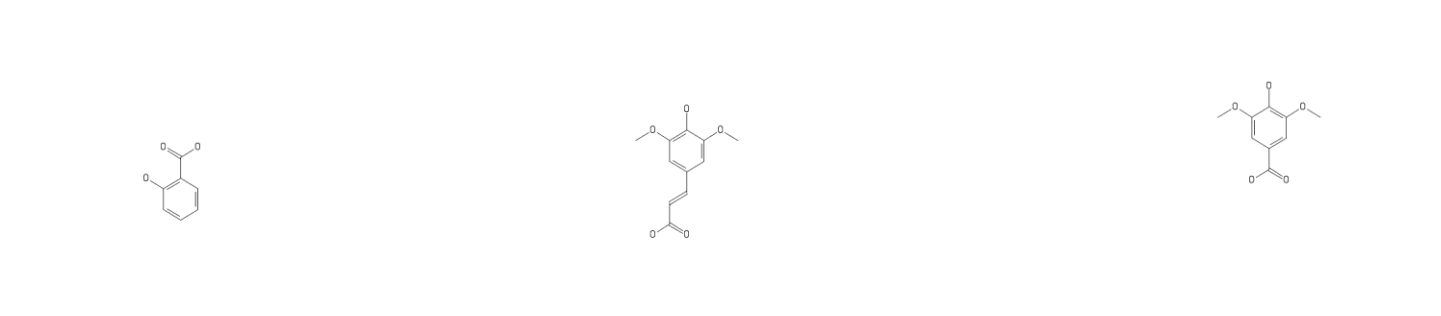


| Salicylic acid | Synapic acid | Syryngic acid |
| --- | --- | --- |
|  |  |  |


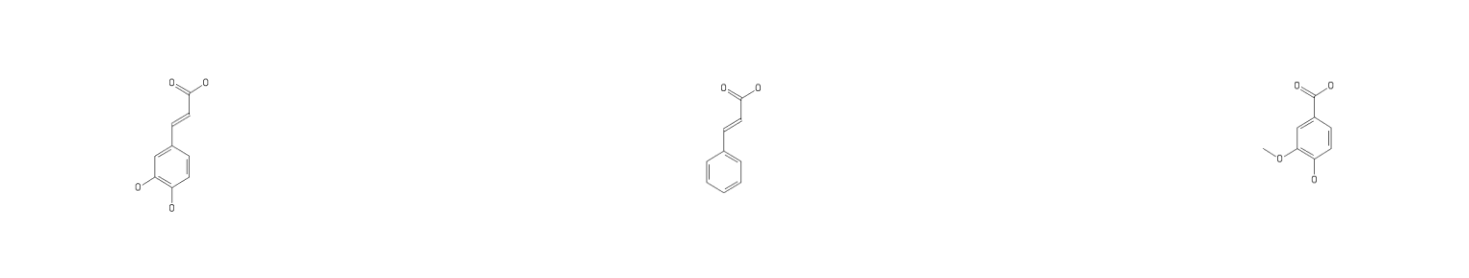


| *Trans* caffeic acid | *Trans* cinnamic acid | Vanillic acid |
| --- | --- | --- |
|  |  |  |
|  | **Phenolic aldehyde** |  |


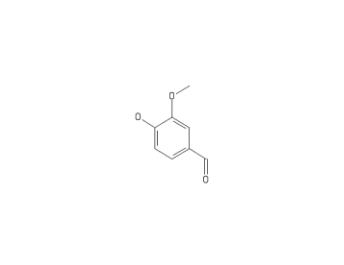


Vanillin


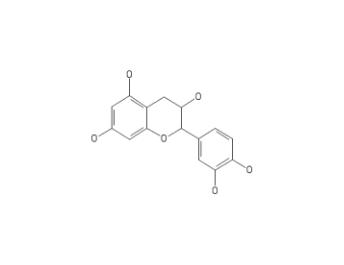


(-)-epicatechin


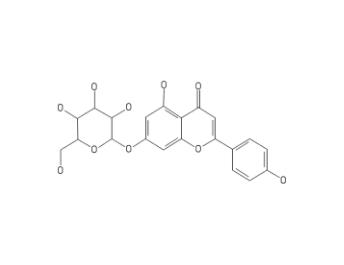


Apigenin 7-*O*-glucoside


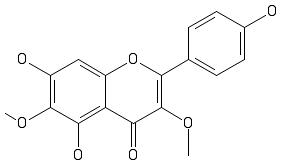


Galetin-3,6-dimethyl ether


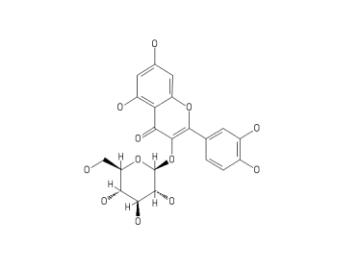


Isoquercetin


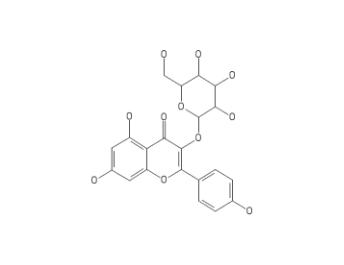


Kaempferol-3-*O*-

glucoside

**Flavonoids**

**
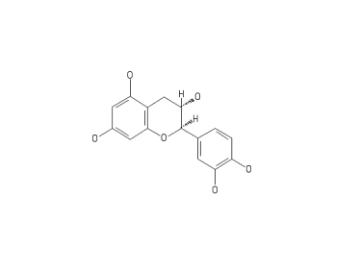

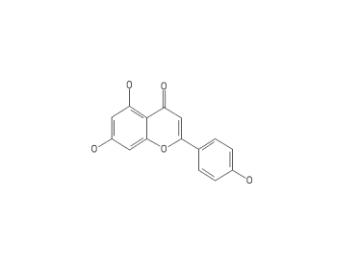
**

(+)-catechin Apigenin


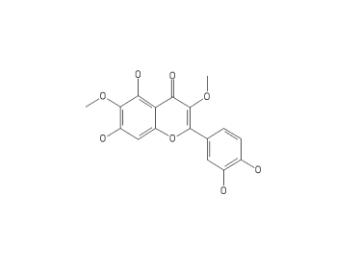

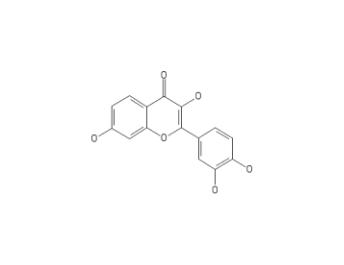


Axillarin Fisetin


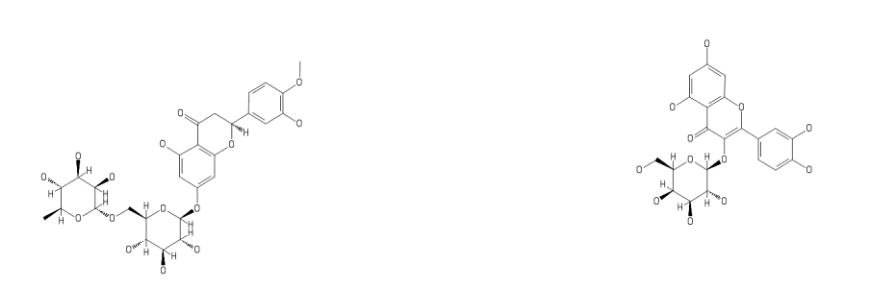


Hesperidin Hyperoside


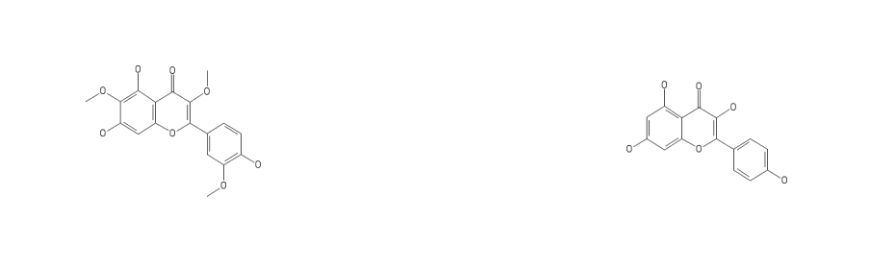


Jaceidin Kaempferol

l


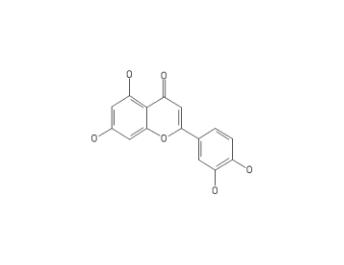

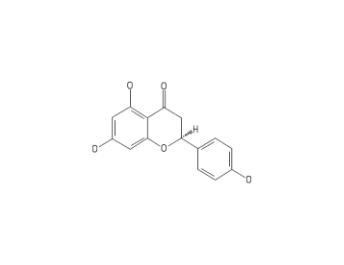


Luteolin Naringeni

n


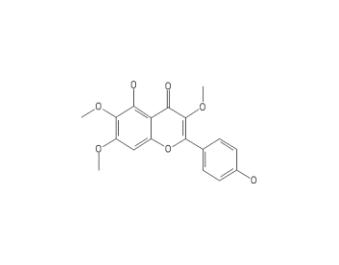


Penduletin


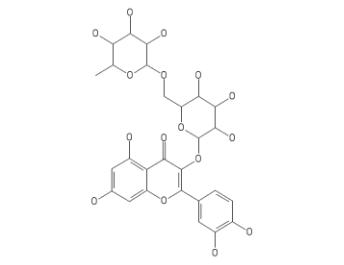


Rutin


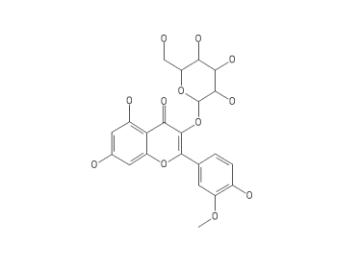


Isorhamnetin-3-*O*-glucoside


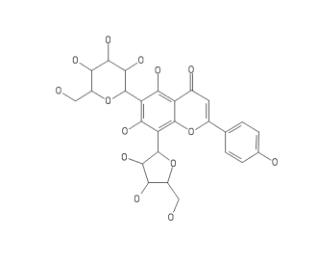


Neoshaftoside


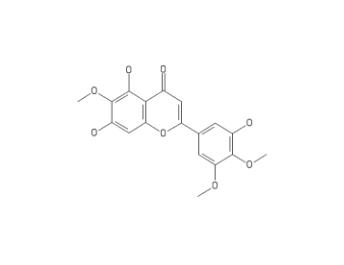


Quercetin


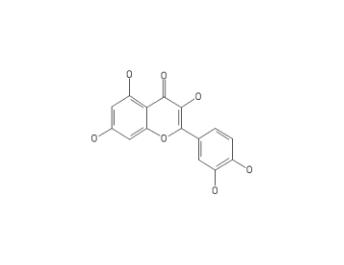


**Flavonoids glycosides**

**Glucosides**

**
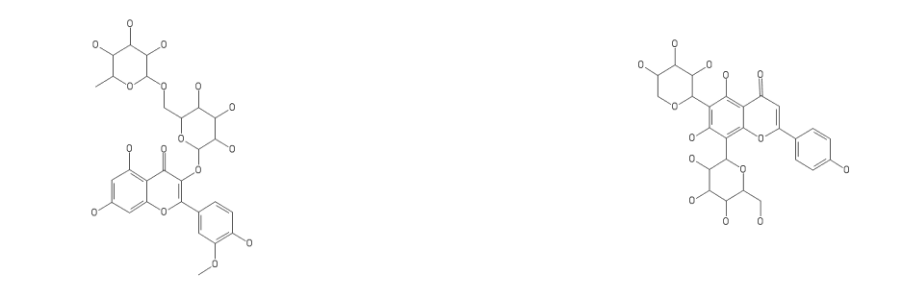
**

Isorhamnetin 3-rutinoside Isoschaftoside


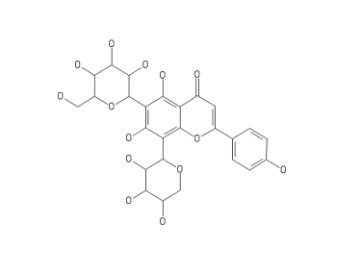


Schaftoside

**Flavonoid aglycones**

**
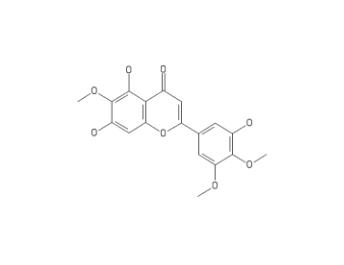

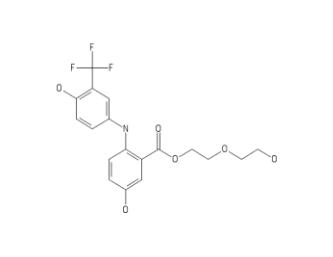
**

| 5,7,3'-triacetoxy- | 5,7,4'-triacetoxy-3,6,4'- | 7-*O*-methyl apigenin |
| --- | --- | --- |
| 3,6,4'-trimethoxyflavone | trimethoxyflavone |  |
|  |  |  |


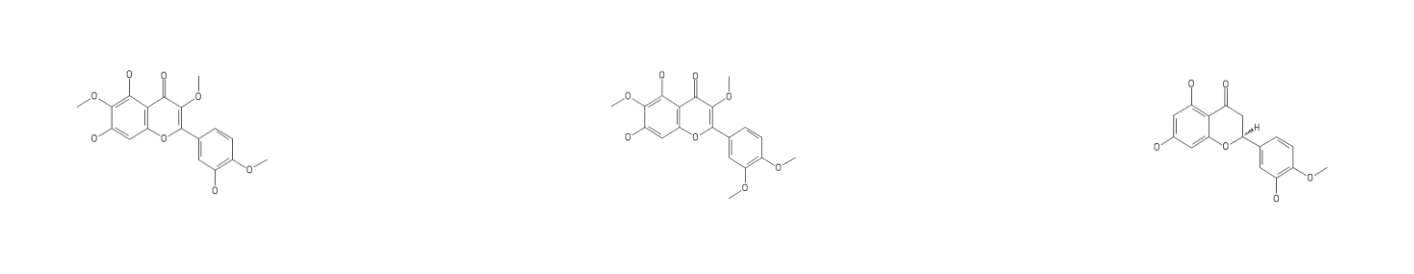


Centaureidin Chrysosplenol B _Hesperetin_

_
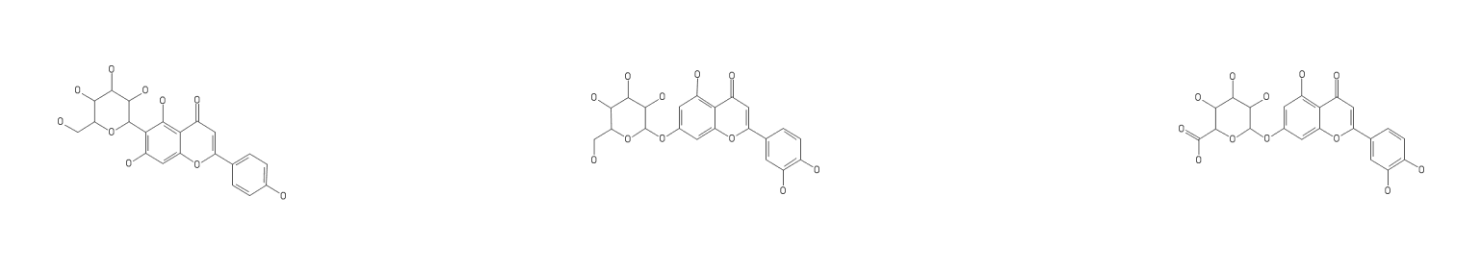
_

Isovitexin Luteolin-7-*O* Luteolin-7-*O*-glucuronide

glycoside


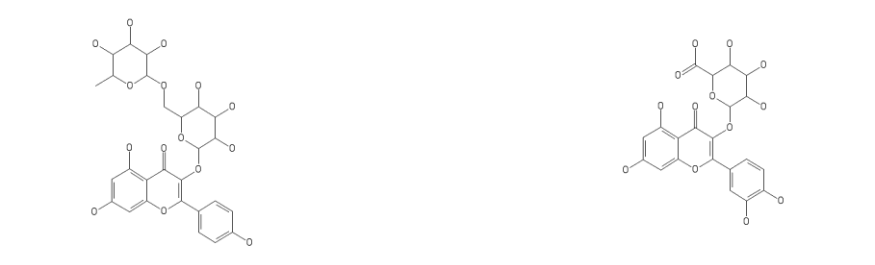


Nicotiflorin Quercetin-3-*O*-glucuronide

**Guaianolides**

**
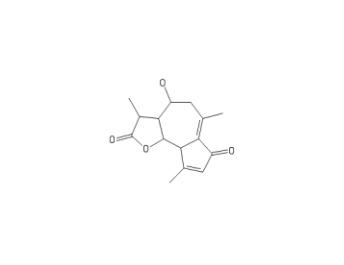

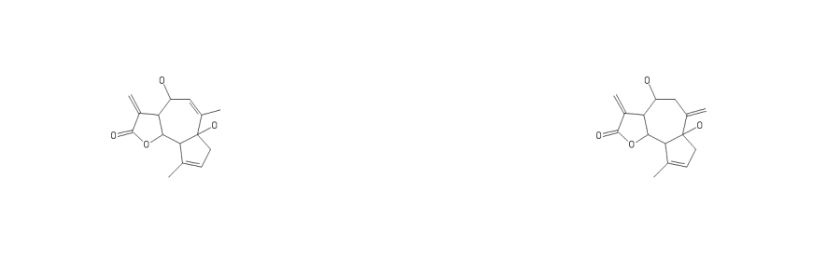
**

1-deoxy-1α-peroxy-rupicolin A 1-deoxy-1α-peroxy-rupicolin B 8-

hydroxyachillin


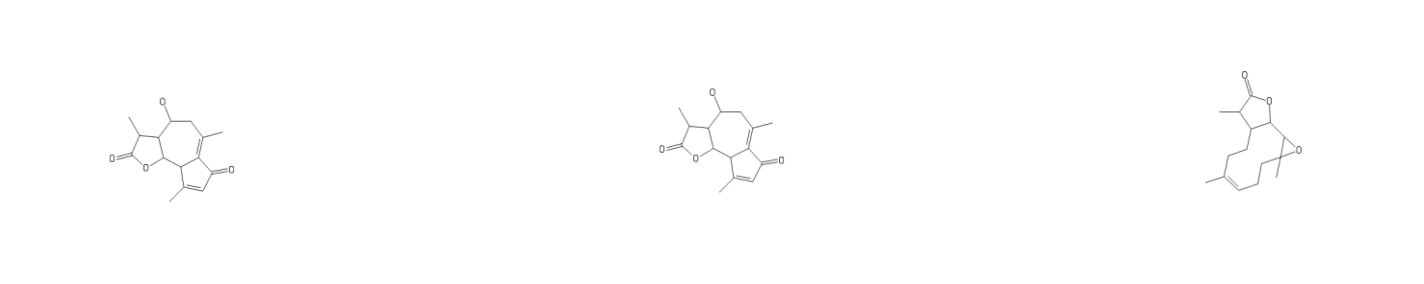


Austricin Desacetylmatricarin Dihydroparthenolide


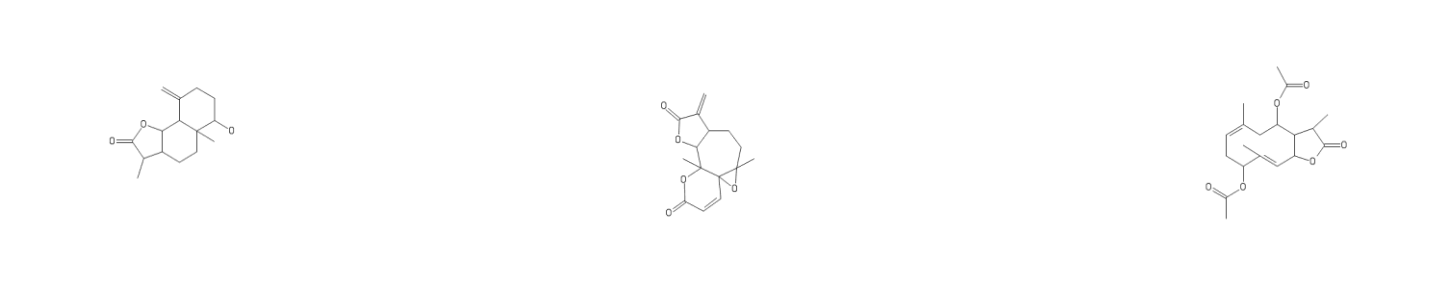


Dihydroreynosin Isopaulitin Millefin


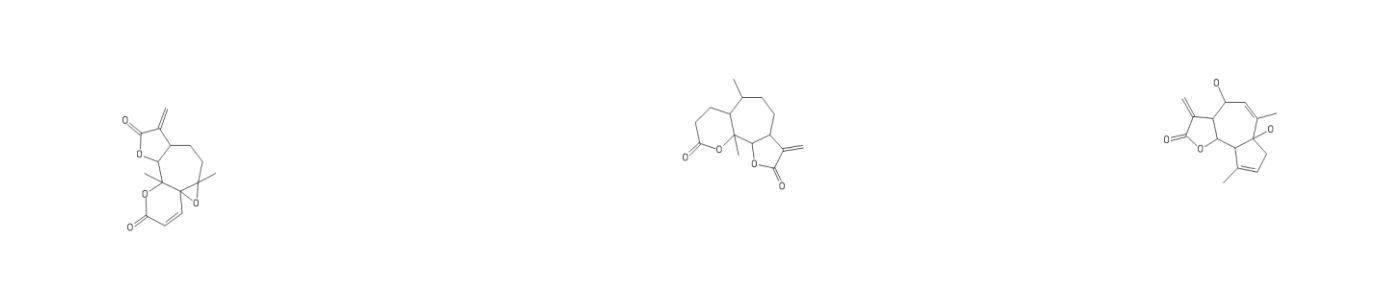


Paulitin Psilostachyin C Rupicolin A


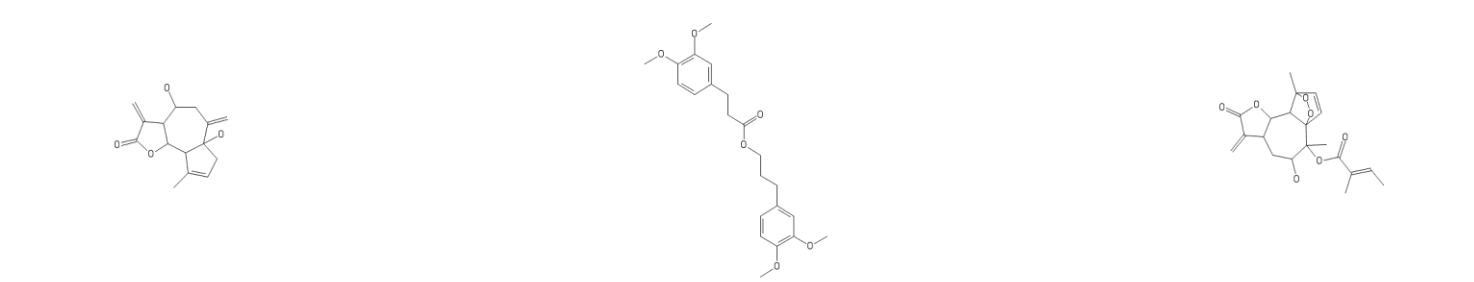


Rupicolin B Sintenin *α*-Peroxyachifolid

**Phytosterols**

**
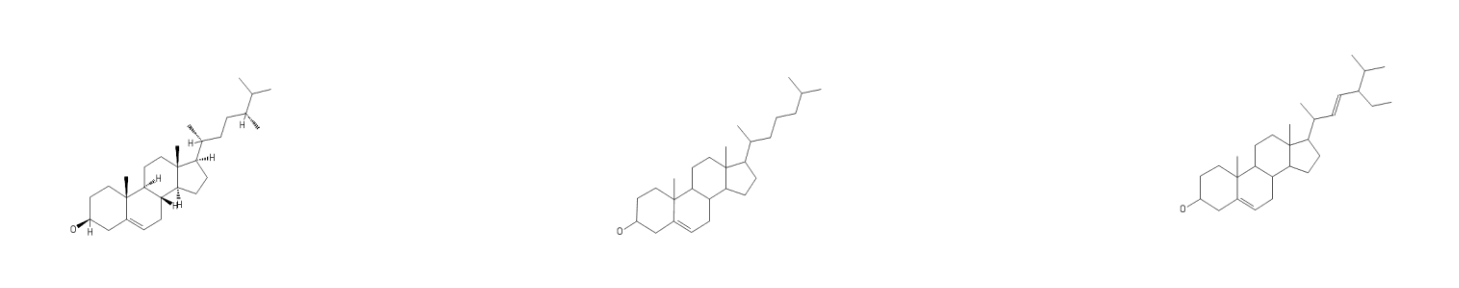
**

|  | Cholesterol | Stigmasterol |
| --- | --- | --- |
| Campesterol |  |  |
|  |  |  |


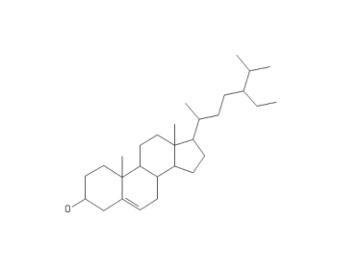


*β*-Sitosterol

**Hydrocarbon monoterpenes**

**
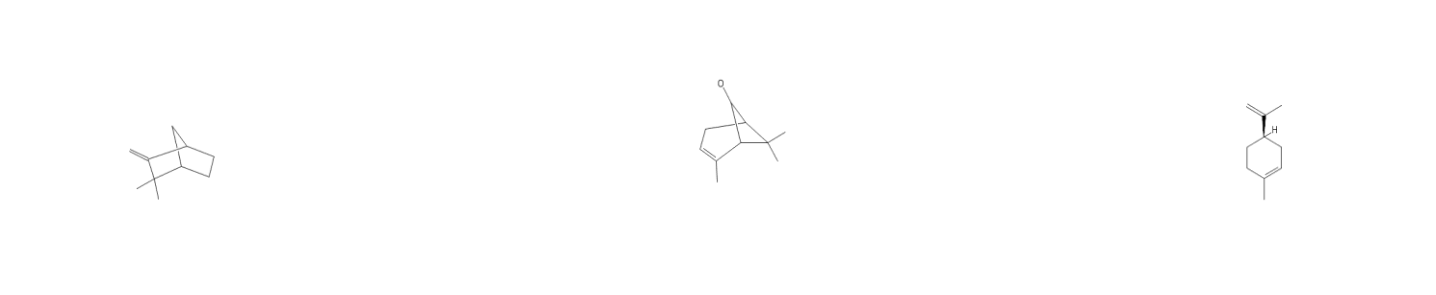
**

^Camphene^ *cis*-Chrysanthenol ^Limonene^

^
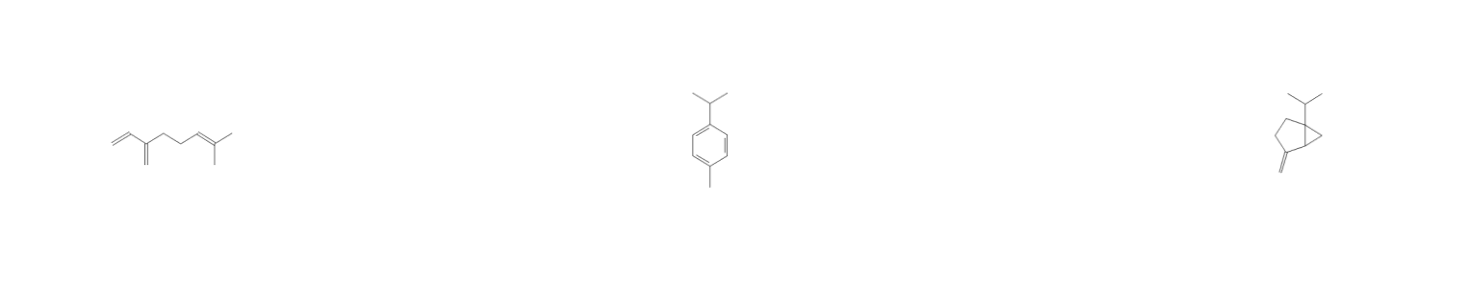
^

Myrcene *p*-cymene Sabinene


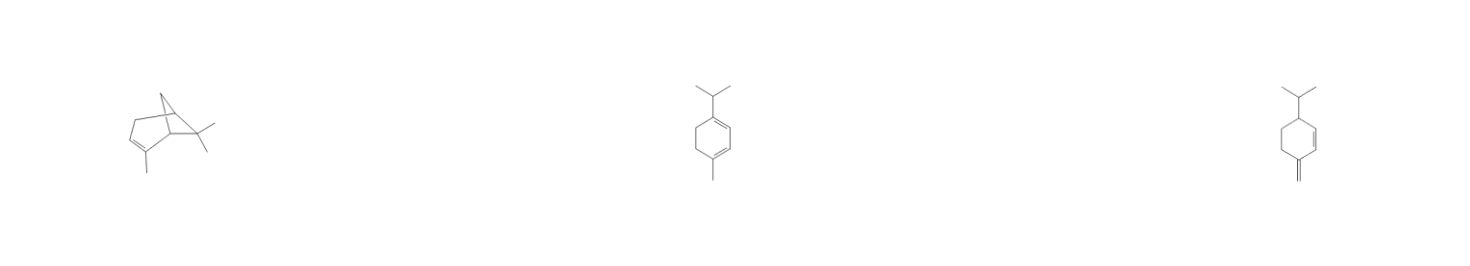


*α*-Pinene *α*-Terpinene *β*-Phellandrene


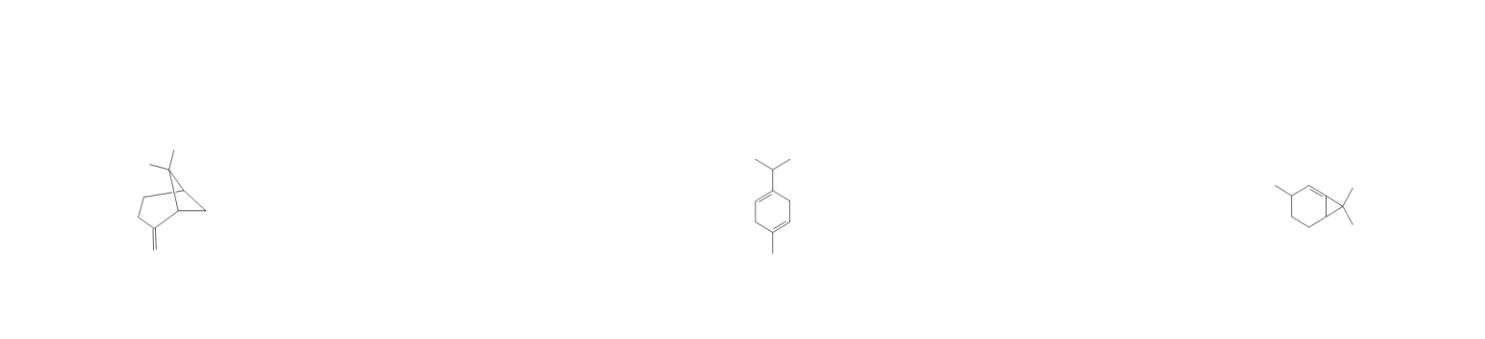


*β*-Pinene *γ*-Terpinene Δ3-Carene

**Oxygeneted monoterpenes**

**
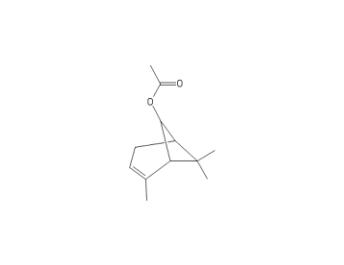

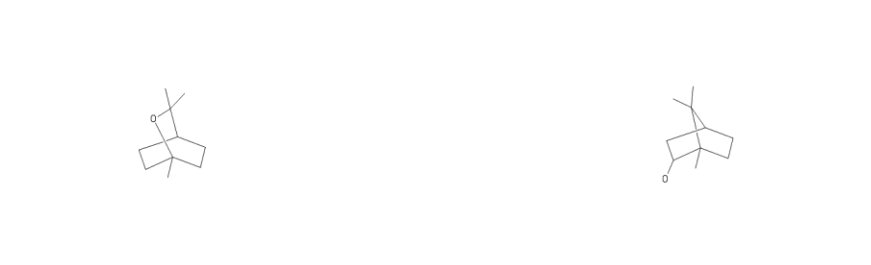
**

*cis*-Chrysanthenyl acetate 1,8-cineole Borneol


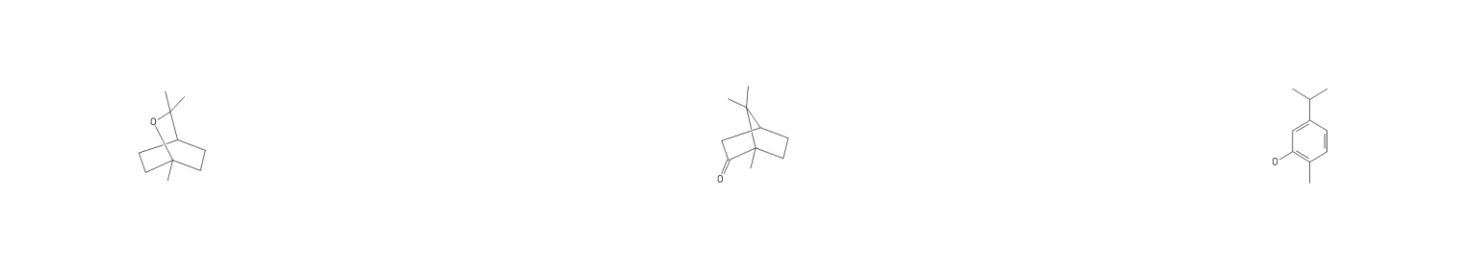


Bornyl acetate Camphor Carvacrol


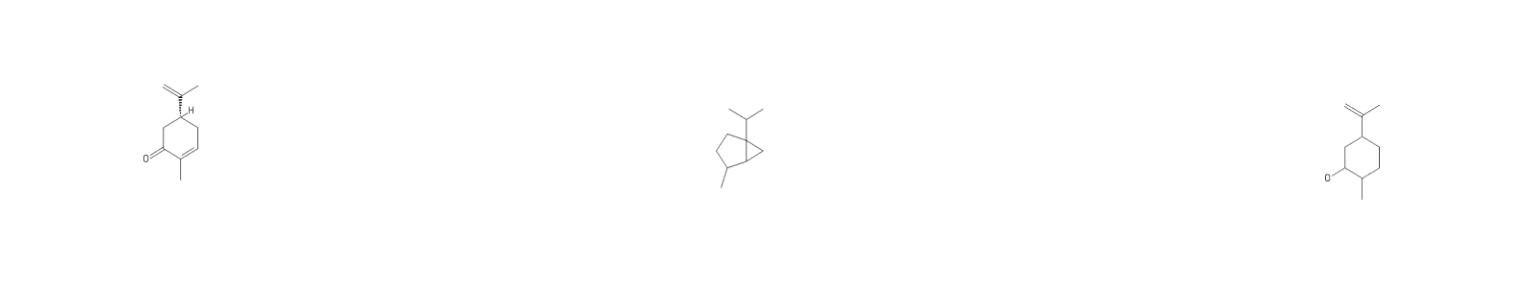


| Carvone | *cis*-Sabinene hydrate | Dihydrocarveol |
| --- | --- | --- |
|  |  |  |


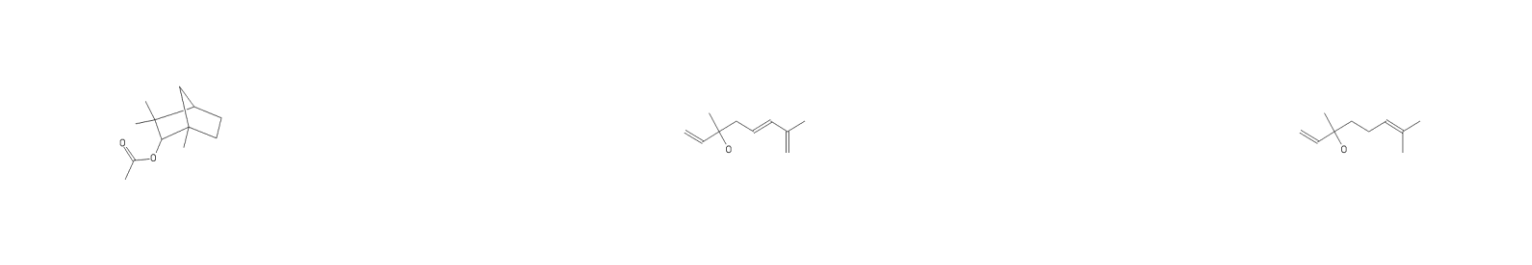


Fenchyl acetate Hotrienol Linalool


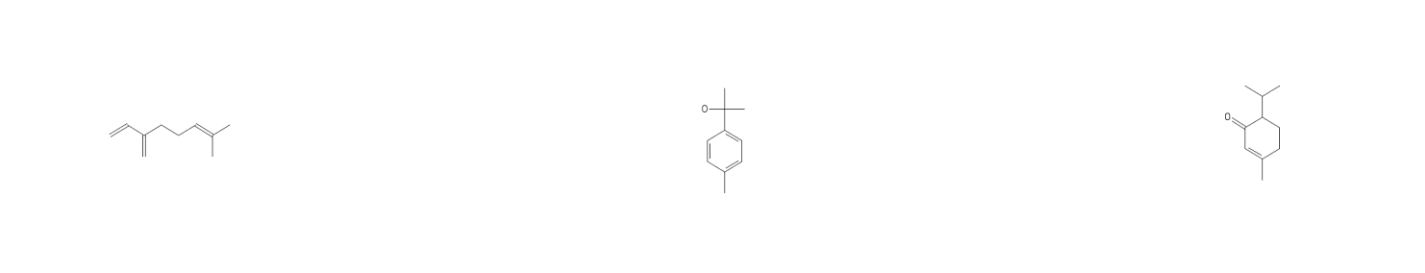


_Myrcene_ *^p^*^-Cymen-8-ol^ Piperitone


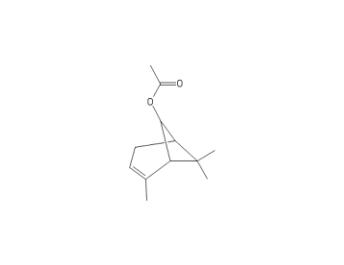

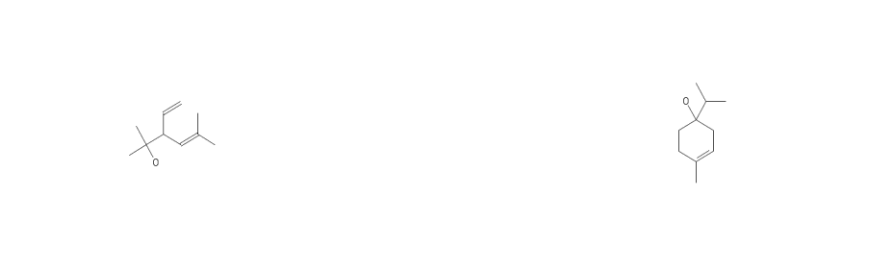


Santolina alcohol Terpinen-4-ol *trans*-Chrysanthenyl acetate


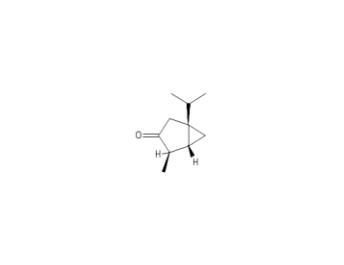

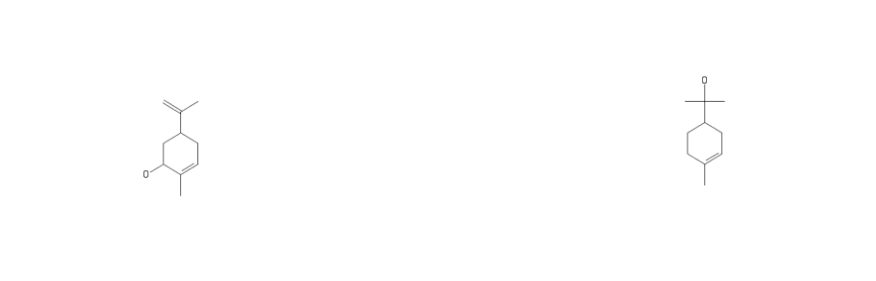


*trans*-iso-Carveol *α*-Terpineol *_α_*_-Thujone_

_
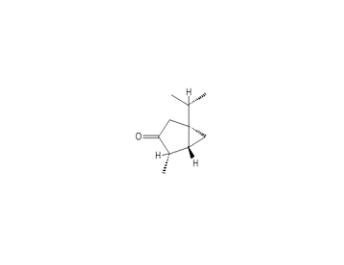
_

β-Thujone

**Diterpenes**

**
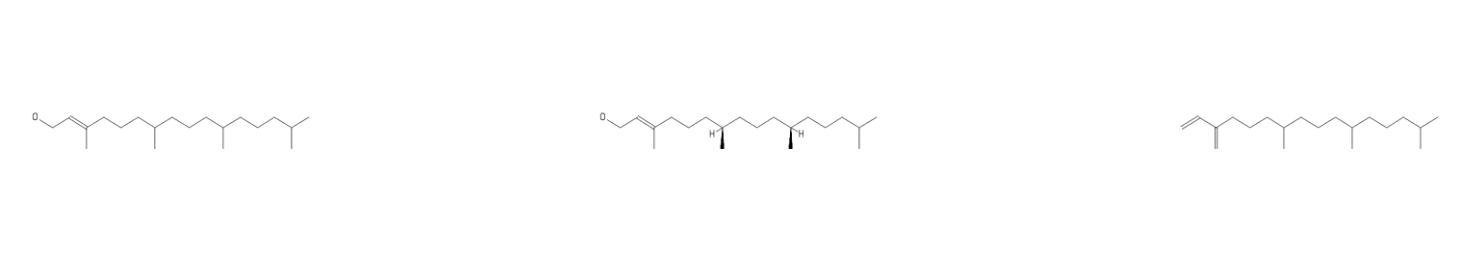
**

(*E*)-Phytol (*Z*)-Phytol Neophytadiene

**Sesquiterpene hydrocarbons**

**
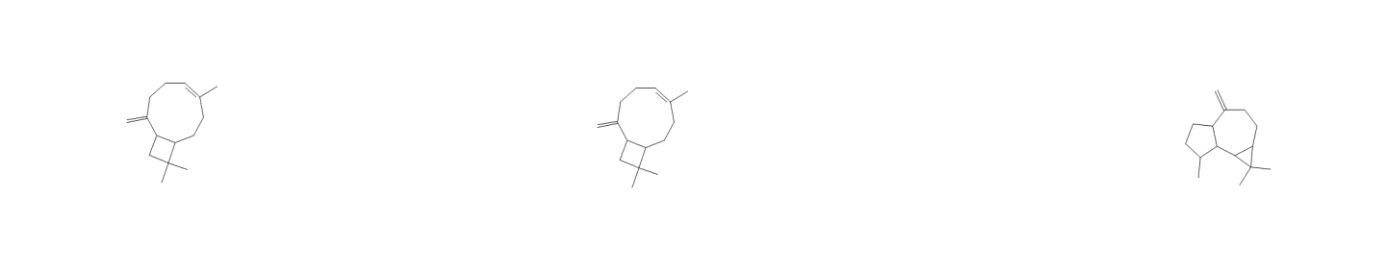
**

(*E*)-Caryophyllene ^(^*^Z^*^)-caryophyllene^ Aromadendrene


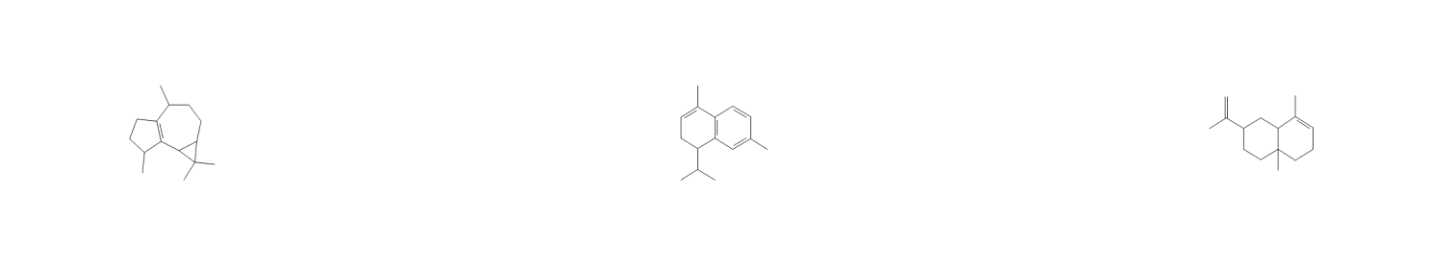


Isoledene α-Calacorene *α*-Selinene


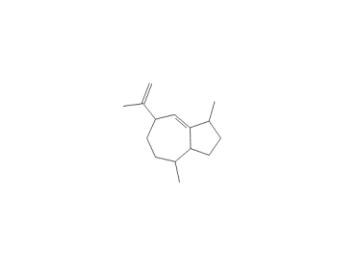


*γ*-Gurjunene

**Oxygenated sesquiterpenes**

**
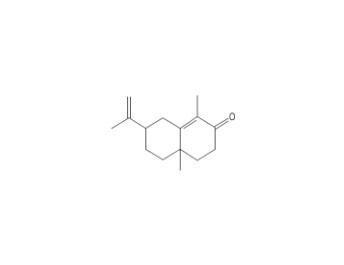
**

*α*-Cyperone

**Sesquiterpenoids**

**
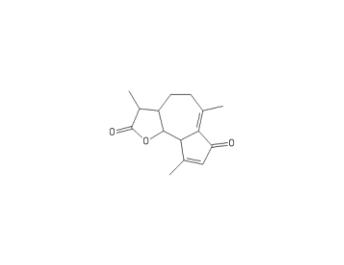
**

Achillin

**Hydrocarbons**

**
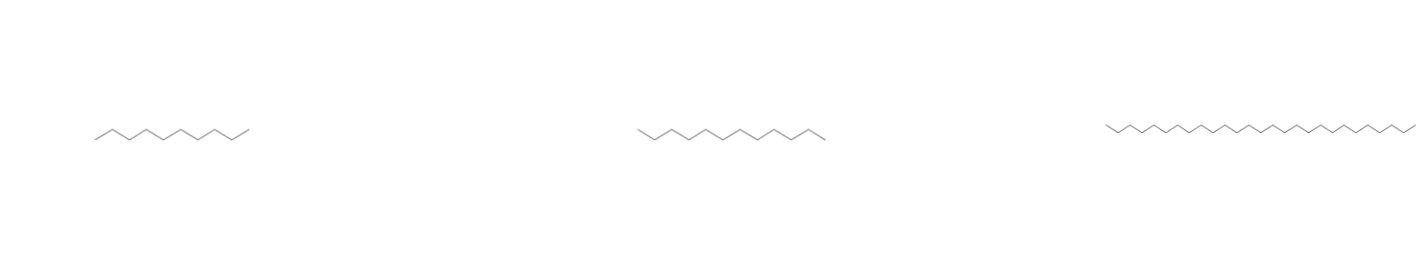
**

Decane Dodecane Heptacosane


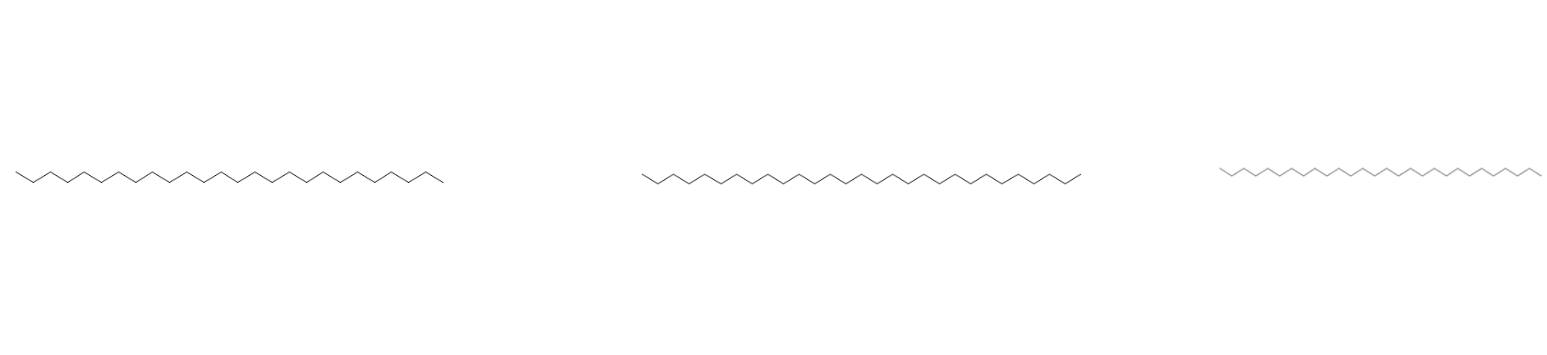


Hexacosane Nonacosane Octacosane


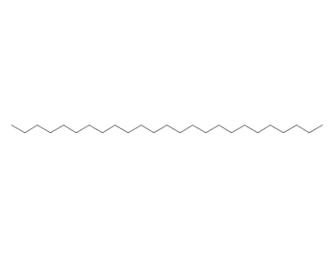

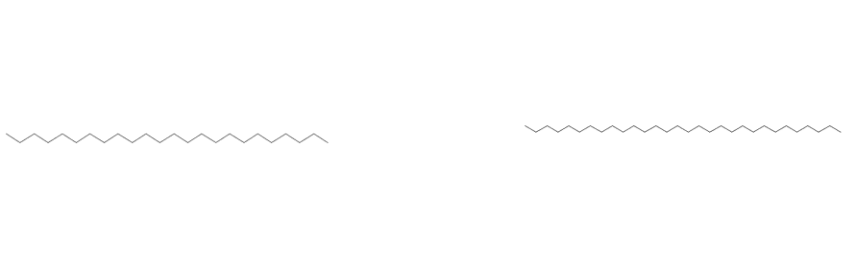


| Pentacosane | Tetracosane | Triacontane |
| --- | --- | --- |
|  |  |  |


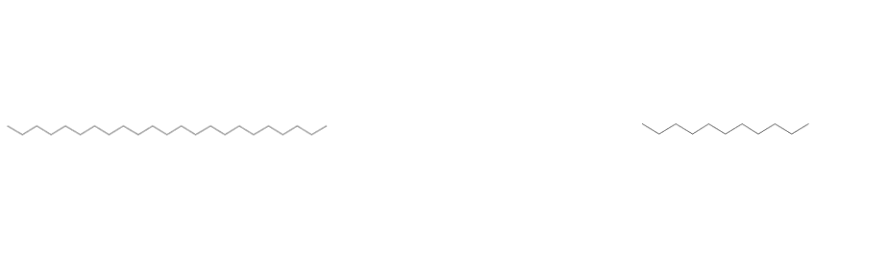


Tricosane Undecane

**Fatty acids and esters**

**
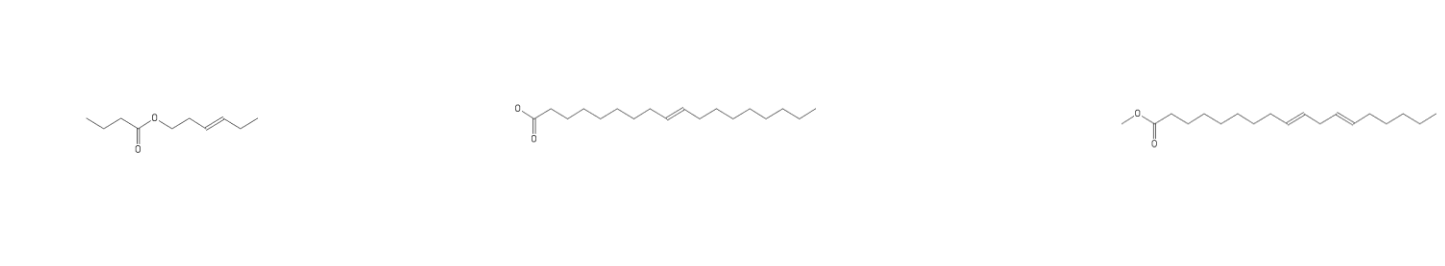
**

| (*Z*)-3-Hexenyl butyrate | (*Z*)-9-Octadecenoic acid | (*Z,Z*)-9,12-Octadecadienoic |
| --- | --- | --- |
|  |  | acid methyl ester |
| 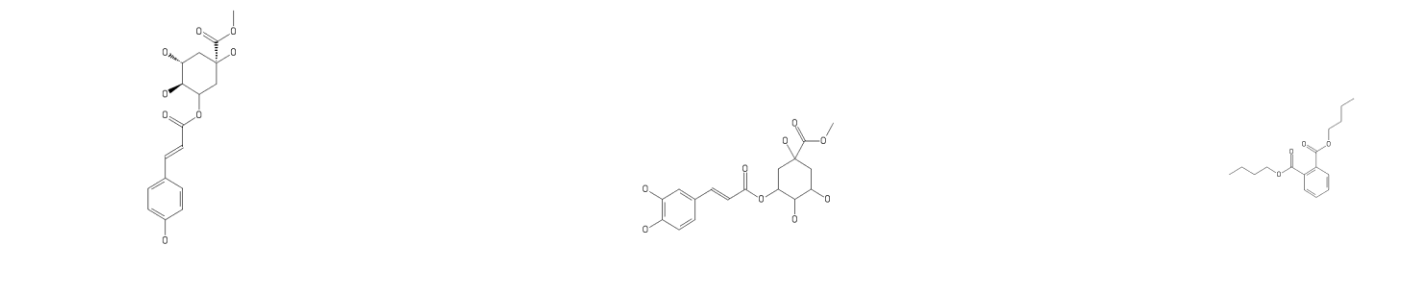 |  |  |

5-*O*-coumaroylquinic

chlorogenic acid methyl ester

acid methyl ester


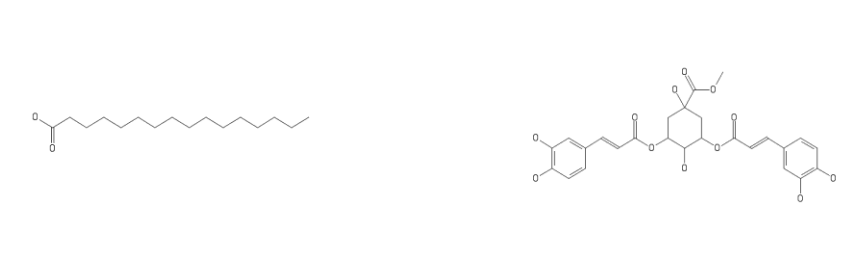


Hexadecanoic acid Methyl 3,5-dicaffeoylquinic acid

**Alcohols**

**
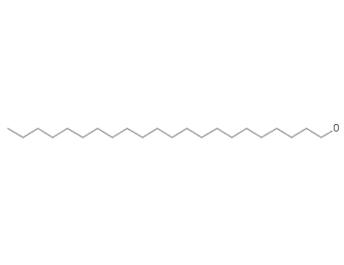

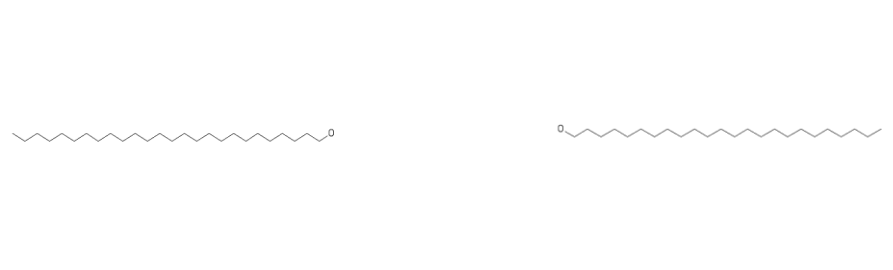
**

Docosanol Hexacosanol Tetracosanol

**Other isoprenoids**

**
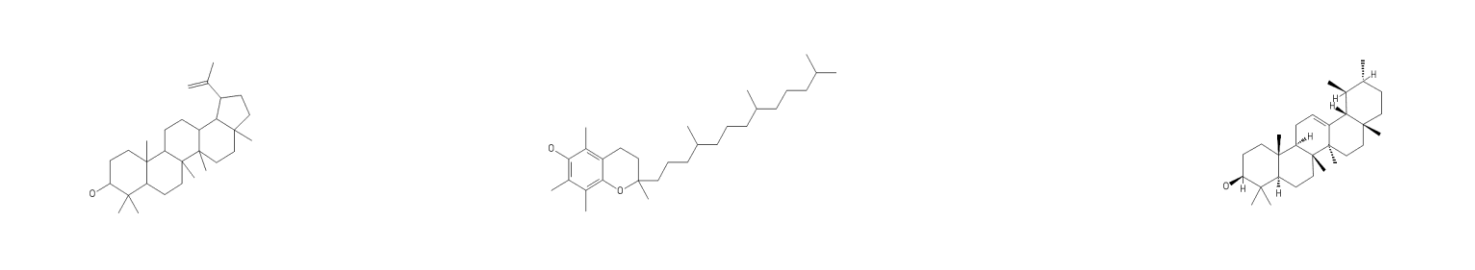
**

| Lup-20(29)-en-3-ol | Vitamin E | *α*-Amyrin |
| --- | --- | --- |
|  |  |  |


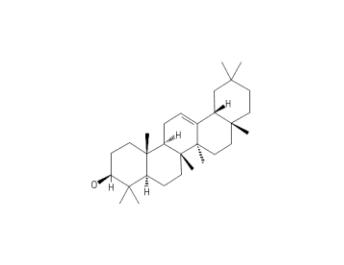


*β*-Amyrin
